# The Vascular Basement Membrane as “Soil” in Brain Metastasis

**DOI:** 10.1371/journal.pone.0005857

**Published:** 2009-06-10

**Authors:** W. Shawn Carbonell, Olaf Ansorge, Nicola Sibson, Ruth Muschel

**Affiliations:** 1 Gray Institute for Radiation Oncology and Biology, University of Oxford, Oxford, United Kingdom; 2 Department of Neuropathology, University of Oxford, Oxford, United Kingdom; Dresden University of Technology, Germany.

## Abstract

Brain-specific homing and direct interactions with the neural substance are prominent hypotheses for brain metastasis formation and a modern manifestation of Paget's “seed and soil” concept. However, there is little direct evidence for this “neurotropic” growth *in vivo*. In contrast, many experimental studies have anecdotally noted the propensity of metastatic cells to grow along the exterior of pre-existing vessels of the CNS, a process termed vascular cooption. These observations suggest the “soil” for malignant cells in the CNS may well be vascular, rather than neuronal. We used *in vivo* experimental models of brain metastasis and analysis of human clinical specimens to test this hypothesis. Indeed, over 95% of early micrometastases examined demonstrated vascular cooption with little evidence for isolated neurotropic growth. This vessel interaction was adhesive in nature implicating the vascular basement membrane (VBM) as the active substrate for tumor cell growth in the brain. Accordingly, VBM promoted adhesion and invasion of malignant cells and was sufficient for tumor growth prior to any evidence of angiogenesis. Blockade or loss of the β1 integrin subunit in tumor cells prevented adhesion to VBM and attenuated metastasis establishment and growth *in vivo*. Our data establishes a new understanding of CNS metastasis formation and identifies the neurovasculature as the critical partner for such growth. Further, we have elucidated the mechanism of vascular cooption for the first time. These findings may help inform the design of effective molecular therapies for patients with fatal CNS malignancies.

## Introduction

Brain metastases are the most common malignant tumors of the central nervous system (CNS) outnumbering primary brain tumors such as glioblastoma in prevalence by tenfold [American Cancer Society, http://www.cancer.org/]. Over 20% of all cancer patients develop metastatic disease to the CNS and have a 9 month median survival with maximal treatment [1]–[4]. As modern therapies allow improved peripheral control of primary and metastatic disease, such as trastuzumab for metastatic breast cancer, the incidence of brain metastasis appears to be paradoxically increasing [4]–[7]. This may be due to several reasons including the blood-brain barrier preventing drug entry, tumor dormancy, positive selection of “brain-seeking” or therapy-resistant subclones, or iatrogenic induction of new functional mutations. Hence, further characterization of these mechanisms and identification of new strategies for treatment of brain metastasis are important goals.

Clinically, brain metastases most commonly arise from lung, breast, and melano-carcinomas. The major requirements for metastasis to distant sites appear to vary by organ and remain incompletely understood [8]–[11]. The pathophysiology of brain metastasis, in particular, remains elusive. In metastasis to lung and bone, characteristic patterns of gene expression in MDA-MB-231-derived mammary carcinoma cells have been shown to enable organ specific colonization [10]. Such factors have not yet been identified for brain metastasis, but are likely to exist as mouse and human carcinoma lines have been selected for increased brain colonization [11], [12]. However, these characterizations of the “seed” largely neglect contributions from the “soil” and appear to be cell line specific [13]. On the other hand, there is a persistent assumption in the literature that brain metastasis is the result of specific interactions with the neural elements of the brain parenchyma mediating “brain homing” (or targeted metastasis to the brain), direct cell attachment and establishment, invasion, and progressive growth into micro- and macrometastases [1], [5], [14], [15]. These ideas are certainly consistent with the classic concept of Pagetian “soil”, however, there currently exist no *in vivo* data to support such statements and indeed very few studies address these topics directly. In contrast, we have noted many experimental brain metastasis studies dating back several decades have anecdotally described early growth of tumor cells along pre-existing brain vessels ([16]–[22]; Table S1). This relationship is reminiscent of vascular cooption described in a rat glioma model [23]. These findings suggest the neural elements of the brain parenchyma do not provide a sufficient substrate for metastatic carcinoma growth and instead implicates the existing neurovasculature as a key niche for malignant progression. This also supports the data by Fidler and colleagues [24] that suggests sprouting neoangiogenesis may not be necessary for the initiation of metastasis growth in the brain.

Here, we used a combination of *in vitro* and *in vivo* studies and human pathological specimens to analyze the temporospatial growth pattern of brain metastasis microcolonies in order to characterize the relationship between metastatic tumor cells and the existing neurovasculature. We focused on timepoints as early as 3d after intravascular injection in order to focus on the earliest events in microcolony formation. We found that brain micrometastases in mouse and human tissue utilized vascular cooption for growth rather than invading and growing within the neural parenchyma. Vascular cooption can be an alternative to neo-angiogenesis and likely acts to deliver blood borne nutrients and oxygen. We propose here that vascular cooption has an additional function for brain metastases; interactions with the pre-existing vessels are required for initial adhesion, proliferation, invasion, and microcolony establishment. We show that the neural parenchyma of the brain cannot substitute in supplying these functions. This work identifies the central role of the vasculature for metastatic growth in the CNS as well as providing insight into the mechanism of adhesive vascular cooption. These novel concepts may allow the development of more effective therapies for brain metastasis.

## Results

### Perivascular growth of early brain micrometastases *in vivo*

**Figure 1:**
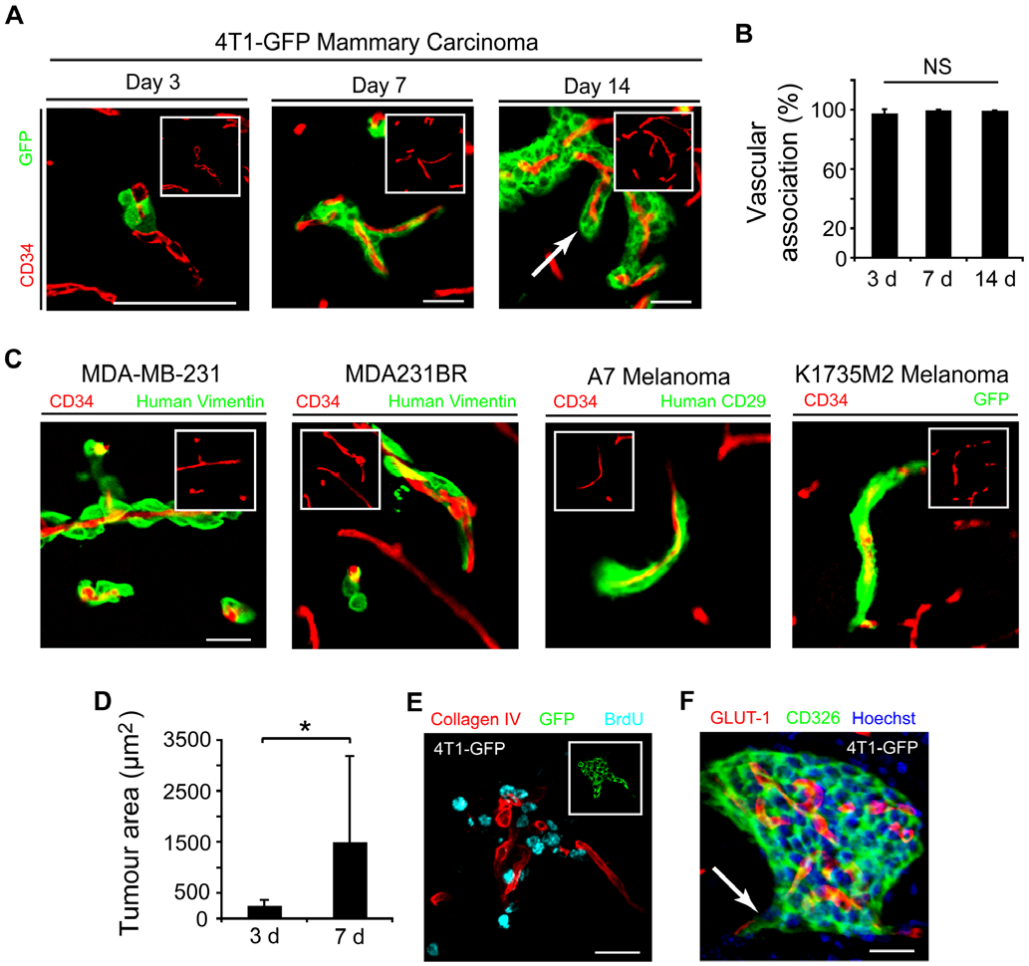
Vascular cooption of cancer cells in brain metastasis formation. (A) Histological analysis of 4T1-GFP microcolonies demonstrating intimate association with CD34+ capillaries (red) from 3 to 14 d after intracardiac injection of cells into syngeneic BALB/c mice. Insets are images of vessels in the absence of the tumor cells/green channel. Arrow shows angiocentric invasion. All scale bars, 30 µm. (B) Quantification of tumor-vascular association of 4T1-GFP at 3, 7, and 14 d (*n* = 3–4 per timepoint). (C) Early brain microcolonies from human cell lines MDA-MB-231, MDA-MB-231BR, and A7 and the murine melanoma K1735M2-GFP demonstrated similar vascular cooption (as indicated). Insets as in (A). Scale bar, 30 µm. (D) Cross sectional tumor area measured at 3 and 7 d demonstrates rapid growth over this time (*P<0.05, *t*-test; *n* = 256 colonies analyzed, 3–4 mice per timepoint). (E) Immunofluorescence for BrdU (cyan) in a tumor colony at 7 d demonstrates a high proportion of proliferative perivascular cells within the colony shown in the inset in green. Scale bar, 30 µm. (F) Projection image of a spontaneous 4T1-GFP brain micrometastasis demonstrates similar features to colonies from the intracardiac model such as Glut-1 positive vessels (red) and angiocentric invasion (arrow). CD326 is an epithelial tumor marker that only stains the tumor cells. Scale bar, 30 µm. Error bars represent s.d.

To characterize the vascular association of tumor cells in experimental brain metastasis models, we examined early brain microcolony formation after intracardiac injection of metastatic mouse and human tumor cells. It has been anecdotally noted that microcolonies in experimental brain metastasis assays often tended to grow along preexisting vessels (Table S1). We established that this pattern occurs with a high frequency and across all cell lines we tested. 4T1-GFP mammary carcinoma cells [25] were found to be intimately associated with the perivascular surface of brain microvessels from the earliest timepoint at 3 d up to 14 d (Fig. 1A) after injection into syngeneic BALB/c mice. This was observed in over 97% of the microcolonies at all timepoints (Fig. 1B). Similar vascular associations resulted from the intracardiac injection of the human breast carcinoma cell lines MDA-MB-231 and its “brain seeking” variant cell line MDA231BR [11], the human melanocarcinoma cell line A7 in SCID mice, and the murine melanoma cell line K1735M2 injected into syngeneic C3H/He mice. Brain microcolonies from each of these cell lines examined between 7 and 14 d after injection were associated with vessels in the same pattern consistent with vascular cooption (Fig. 1C). Interestingly, the “brain seeking” MDA231BR line showed equivalent vascular association and microcolony area as the parental line, although the number of colonies produced by injection of the parental cells was approximately 2 fold less (Fig. S1). Thus perivascular colony formation was the predominant pattern of growth by carcinoma cells in experimental *in vivo* metastasis assays.

We verified that the colonies resulted from proliferation of vascular-associated tumor cells between 3 and 7 d by measuring tumor area (Fig. 1D) and with BrdU immunohistochemistry (Fig. 1E). Similar to prior studies [16], [24], [26], these microcolonies appeared to rely on pre-existing vessels for growth. First, proliferation of tumor cells was observed within 1 week of injection and prior to any evidence of neoangiogenesis (Figs. 1D and 1E). Second, vessel morphology appeared largely normal, however, vessel density was significantly lower in tumor-involved areas of the brain (Fig. S2). Third, despite the extensive perivascular involvement of the tumors, the endothelial blood brain barrier (BBB) markers GLUT-1 (Fig. 1F and S2) and ZO-1 (not shown) remained unaltered. We verified BBB integrity with static and dynamic studies of function including gadolinium-enhanced MRI (Fig. S2), small molecular weight TRITC-dextran injection (not shown), and tyramide-signal amplification enhanced immunohistochemistry for IgG extravasation (Fig. S2) at timepoints up to 14 d. These results demonstrated that early brain micrometastasis formation was associated with minimal neoangiogenesis leading to the conclusion that growth must be dependent upon pre-existing vessels. Finally, to verify the perivascular preference of brain micrometastases was not biased by intravascular delivery of tumor cells, we characterized a syngeneic model of spontaneous brain metastasis [27]. Brain sections were examined for spontaneous micrometastases 5–7 weeks after orthotopic injection of 4T1-GFP cells into the mammary fat pad (*n* = 10 mice). The growth and morphological characteristics of these colonies were indistinguishable from those derived from intracardiac delivery of cells demonstrating both intact GLUT-1-positive vasculature and angiotropic spread upon adjacent capillaries (Fig. 1F).

**Figure 2:**
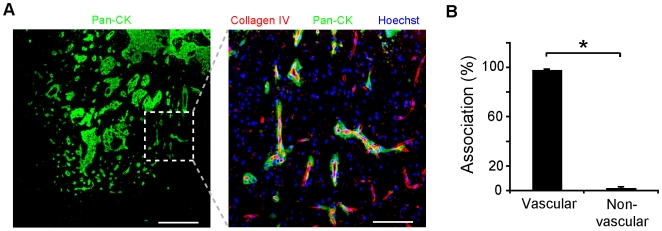
Vascular cooption in human brain metastasis. (A) Human carcinomatous metastasis from lung primary tumor (third case in Table 1) demonstrates vascular association. Right, high power view of hatched region. Scale bars, 0.5 mm (left), 120 µm (right). (B) Quantitation of vascular association in human brain metastasis specimens of diverse primary origin from six patients (*P<0.001, t-test, *n* = 3420 tumour profiles from 6 patients; Table 1). Error bars represent s.d.

To assess the clinical relevance of frequent vascular cooption in experimental metastasis, we asked whether tumor cells within human brain metastasis specimens displayed a similar vascular association. In brain micrometastases and tumors with carcinomatous CNS spread that could be considered at the early stages of parenchymal colonization, the patterns were similar to those in the experimental models above, with the tumor cells in these brain metastases appearing to track along the blood vessels (Fig. 2A). Quantitation of vascular cooption in these cases from primary tumors of varied origin revealed that 98.2% of metastatic brain colonies were vascular-associated (Fig. 2B and Table 1). Based on clinical pathologic indices, there was little clear morphological evidence characteristic of tumor angiogenesis in these cases (Table 1 and Fig. 2A). Therefore, early growth of brain metastasis microcolonies in experimental models and human clinical specimens occurs via intimate interactions with the existing neurovasculature. This growth can occur immediately after extravasation (as early as 3 d after intra-arterial injection) and does not require neovascularization.

**Table 1:** Brain metastases are highly associated with pre-existing vessels in human clinical specimens.

Primary	Growth pattern	Number profiles	Single cell (%)	Vascular associated (%)	Vascular ectasia*	Endothelial hypertrophy*	Multi-layer vessels*
Breast	V-R	338	166 (49.1)	337 (99.7)	no	no	no
Lung NSC	micro/sol	430	406 (94.4)	419 (97.4)	very rare	very rare	no
Lung NSC	V-R	1000	684 (68.4)	985 (98.5)	very rare	rare	no
? Breast, gyn	V-R	1000	788 (78.8)	982 (98.2)	very rare	rare	no
? Breast	V-R	393	309 (78.6)	386 (98.2)	very rare	no	no
Melanoma	micro	264	256 (97.0)	256 (97.0)	no	rare	no

* Clinical indices of angiogenesis.

Abbreviations: lung NSC, lung non-small cell carcinoma; gyn, gynecological carcinoma; micro, micrometastasis; sol, solitary metastases; V-R, Virchow-Robin space invasion.

### Vascular cooption after intraparenchymal injection

**Figure 3:**
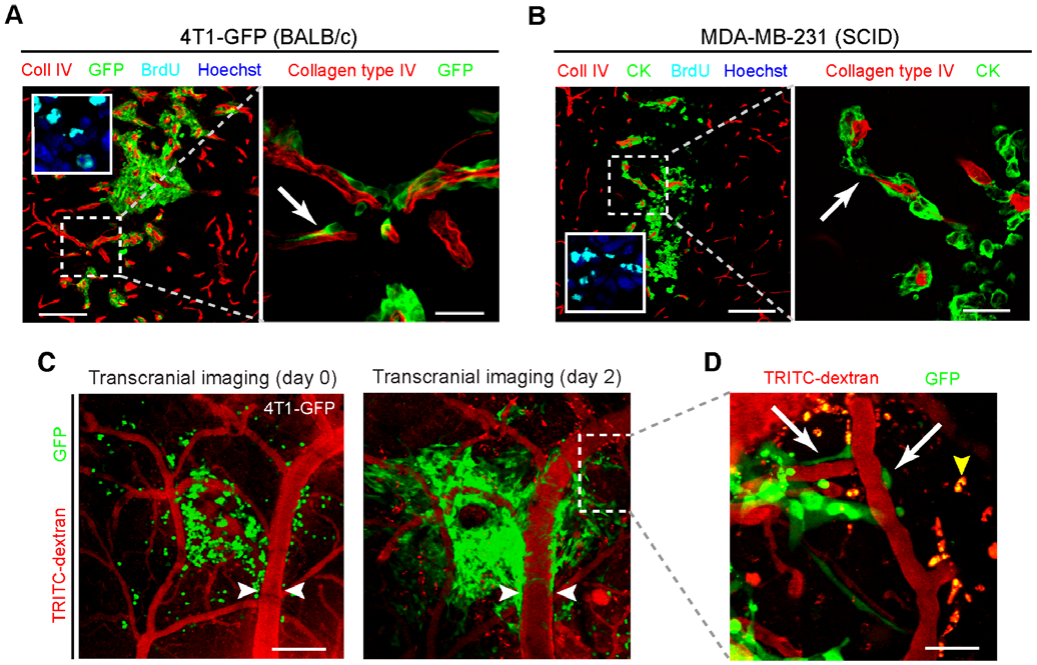
Vascular preference for CNS growth of carcinoma cells *in vivo*. 10^4^ (A) 4T1-GFP or (B) MDA-MB-231 cells were injected directly into the forebrain of mice. Representative coronal sections are shown after 4 d and demonstrate angiotropic growth (left; inset, BrdU from the main tumor mass on an adjacent section). The right panels show a magnification of the hatched areas on the left further documenting the interaction of the tumor cells with the vessels, in this case angiocentric invasion (arrows). Scale bars, 120 µm (left), 30 µm (right). (C) Serial transcranial confocal/multiphoton imaging through a cranial window showed that colony expansion occurred along pre-existing vessels *in vivo* (*n* = 5 mice). Day 0 (left), Day 2 (right). Note dilatation of coopted vein compared to day 0 (white arrowheads). Scale bars, 240 µm. (D) Detail of hatched area in (C) demonstrates tumor cells now present in previously uninvolved areas with extensive spreading on pre-existing vessels (arrows). Yellow arrowhead, TRITC-dextran laden phagocytes. Scale bars, 30 µm.

We further asked whether the association of tumor cells with the vessels was merely a matter of location after extravasation. This was tested by injecting tumor cells directly into the brain allowing equivalent access to both the vascular and the neural elements of the brain. 4T1-GFP cells after intraparenchymal injection formed colonies surrounding vessels within 2 days (Fig. 3A). 93% of tumor profiles were vascular-associated at 4 d (4T1-GFP cells, *n* = 3). The apparent increase in the number of tumor cells could not be simply due to migration and homotypic aggregation of the injected tumor cells because BrdU labeling showed widespread proliferation of the tumor cells (Fig. 3A, inset). Similar results were obtained after intraparenchymal injection of MDA-MB-231 cells (Fig. 3B). Thus, vascular association or cooption does not rely on cells entering the brain via extravasation from the blood supply and must be due instead to preferential association of the tumor cells with the vessels.

To further these findings, we directly observed tumor cells *in vivo* using transcranial multiphoton and confocal imaging through a cranial window in mice. This preparation allowed us to serially revisit the same fields through the cranial window from 0 to 4 days following intraparenchymal injection. Injected tumor cells were seen to elongate upon blood vessels within 1 hour of injection (Fig. S3). The majority of recently injected cells were spheroidal; after 2 d there was an obvious expansion of tumor cells in the same field (Fig. 3C). They appeared to be exclusively growing upon vessels (Figure 3D). The vascular pattern was not noticeably modified after 2 days and there was no appearance of new vessels consistent with tumor growth dependent upon preexisting vessels. However, some vascular ectasia was noted and possibly associated with greater tumor burden and/or a response to the tissue disruption due to the injection (Fig. 3C, white arrowheads). These histological and transcranial imaging studies were repeated using direct injection of B16F10-GFP mouse melanoma cells with similar results (Fig. S3). On the basis of these studies, we conclude that the vascular association of tumor cells in brain colonies is not simply due to the physical association of the tumor cell with the vessel after extravasation, but is based upon preferential interactions with the vessel.

### Vascular cooption occurs by direct adhesion to vessel wall exterior

**Figure 4:**
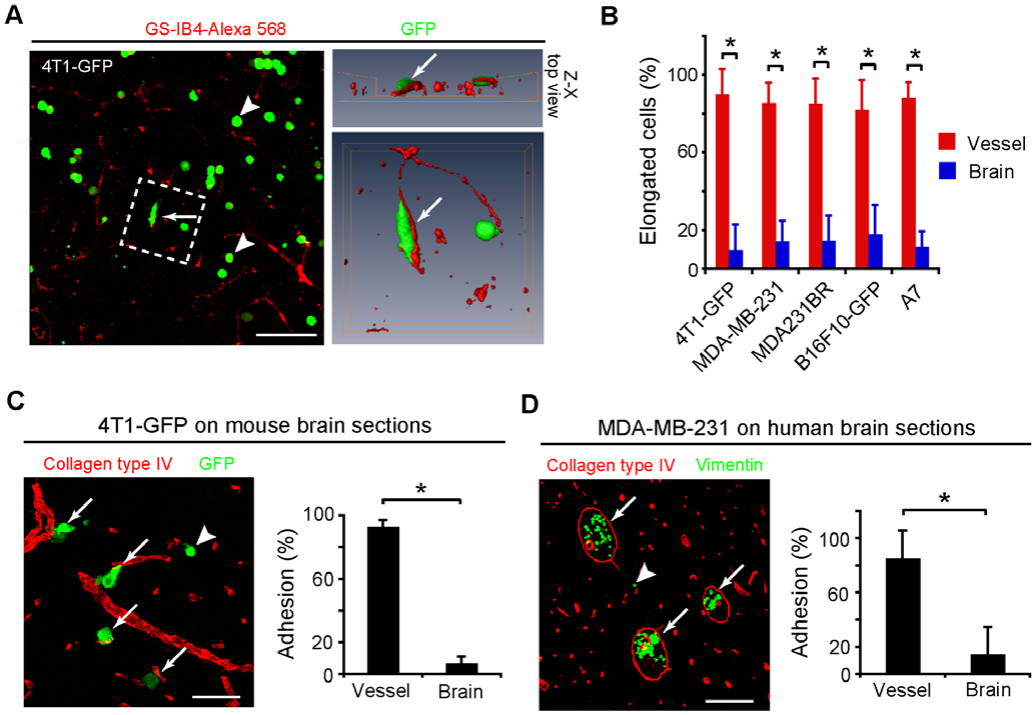
Tumor cell interactions with CNS vessels. (A) 4T1-GFP cells were plated onto live adult murine brain slices and fixed after 2 h of co-culture. 3-D reconstructions of hatched area (right panels) verify that the elongated tumor cell is spreading upon a vessel (arrows). Scale bar, 120 µm. (B) Quantitation of vascular association on live brain slices with several mouse and human tumour lines demonstrates the vast majority of elongated cells (82–90%) are attached to vessels (*P<0.05, Mann-Whitney U-test, *n* = 294–546 cells per line, 2 independent experiments in duplicate). (C) 4T1-GFP cells were plated onto slide-mounted tissue sections of murine brain for 2 h and non-adherent cells were rinsed off. Tumor cells preferentially attached to vessels (arrows) rather than the neural elements (arrowhead). Graph, the location of the adherent cells was assessed (*P<0.01, Mann-Whitney U-test, 2 independent experiments in triplicate). Scale bar, 60 µm. (D) Human MDA-MB-231 cells (arrows) were plated on slide-mounted human brain sections. They similarly attached to vessels preferentially (arrows) over the neural parenchyma (arrowhead). (*P<0.01, Mann-Whitney U-test, *n* = 3 patient samples, 2 independent experiments in duplicate). Scale bar, 240 µm. All error bars represent s.d.

We sought to generate experimental situations in tissue culture analogous to the intraparenchymal injections to characterize the interaction of metastatic tumor cells with the vascular or neural elements of the brain. First, we used an *ex vivo* assay with tumor cells plated onto live, acutely isolated adult mouse brain slices [28]. All five of the murine and human tumor lines tested for experimental brain metastasis in Fig. 1 were plated on live slices. Each of these cell lines was found to preferentially elongate upon the vasculature within 2 hours (Fig. 4A, 4B and S4). Tumor cells not directly in contact with vessels did not spread (Fig. 4A and S4). These results were directly verified with timelapse confocal microscopy (Movie S1). Thus, we concluded that metastatic carcinoma cells, when given equal access to live vascular and neural substrates, interact preferentially with the vessels over the neural parenchymal elements.

Based on morphological features of cells and microcolonies in histological sections (Figs. 1 and 2) and the rapid cell spreading on live slices observed above, we reasoned that there was likely an active adhesive interaction between the metastatic tumor cells and the exterior of blood vessels. To test the potential for tumor cell adhesion to elements of the brain parenchyma, either vascular or neural, we assayed adhesion of metastatic tumor cells plated on thawed slide-mounted snap frozen brain sections. 4T1-GFP cells were plated on normal murine brain and cultured for 2 h followed by washing off non-adherent cells. Only a small minority of plated cells adhered to the slices, however, 93% of these adherent cells were in contact with vessels (Fig. 4C). To test the adhesion of human tumor cells, we used the MDA-MB-231 breast carcinoma line in a parallel assay with human brain sections. Similarly 85% of the adherent human cells were associated with vessels (Fig. 4D and S5). These results demonstrated that metastatic tumor cells adhere to the brain parenchyma and that their preferred substrate is vascular rather than neural. These studies complement the observations in live slices that showed tumor cell spreading to be associated with vascular, not parenchymal engagement.

### Vascular cooption promotes CNS invasion of metastatic cells

**Figure 5:**
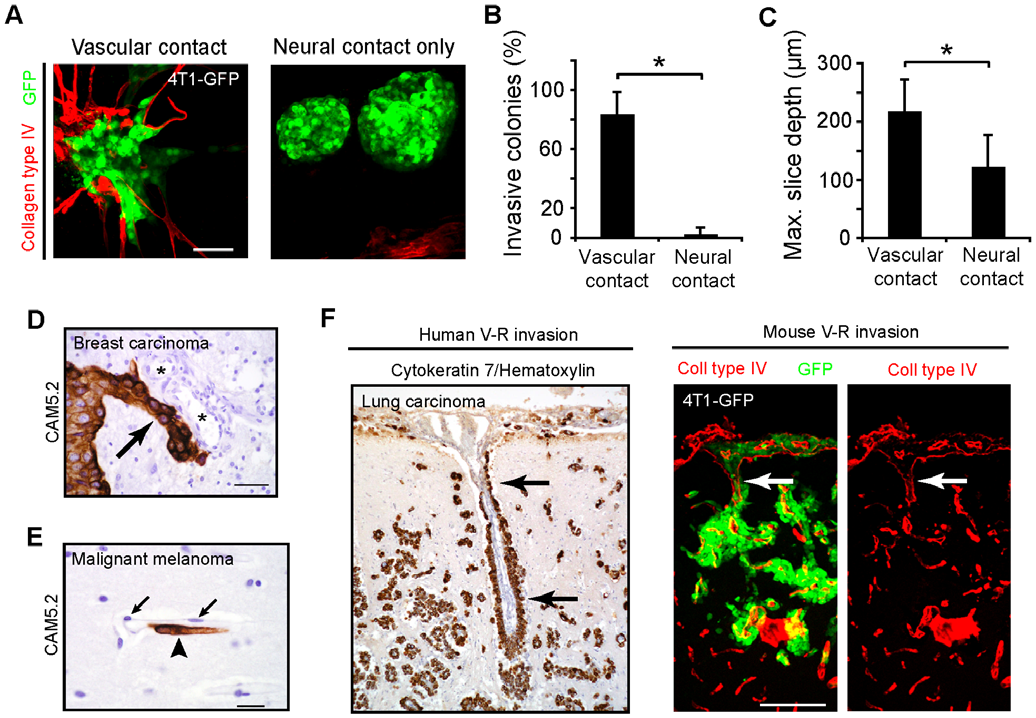
Angiocentric invasion of parenchyma by brain metastases. (A) 4T1-GFP spheroids were plated onto live brain slices to assess parenchymal invasion. 4T1-GFP spheroids invaded live brain slices upon the vascular scaffolding (left), but those spheroids contacting the brain parenchyma in regions without vessels showed little morphological evidence of invasion (right). Scale bar, 60 µm. (B) The percent of invasive colonies associated with vessels was significantly higher than those in contact only with the brain (*P<0.05, Mann-Whitney U-test, 2 independent experiments). (C) The maximum depth of invasion by each spheroid into the brain slice was measured further confirming a vascular preference (*P<0.001, Mann-Whitney U-test, 2 independent experiments). (D) Example of collective angiotropic invasion (arrow) from a human specimen of brain metastasis from breast carcinoma. Asterisks, vessel lumens. Scale bar, 50 µm. (E) Example of single-cell angiocentric invasion (arrowhead) in the brain from a human specimen of malignant melanoma brain metastasis. Arrows show endothelial cell nuclei. Scale bar, 20 µm. (F) Left, angiocentric parenchymal invasion (arrow) was observed to occur from the Virchow-Robin spaces in human cases with carcinomatous CNS spread (lung carcinoma, scale bar, 100 µm) as mirrored in the 4T1-GFP mouse model, right (scale bar, 120 µm). All error bars represent s.d.

The preferential vessel adhesion and spreading of metastatic tumor cells raised the possibility that invasion might also depend upon the vasculature. To determine whether metastatic tumor invasion required vessels or could occur via neural elements, we modeled vascular invasion *in vitro* by plating 4T1-GFP spheroids on live post-natal brain slices. After 3 to 7 days of co-culture, spheroids invaded the slice almost exclusively upon the vascular scaffolding (Fig. 5A and 5B). Spheroids that failed to contact vessels showed little evidence of invasion into the brain slice (Fig. 5A, right). The importance of vascular contact for invasion was confirmed by measuring the depth of penetration into the slices. The spheroids in contact with the vessels penetrated significantly deeper into the brain slice than those without contact (Fig. 5C). Thus, invasion of carcinoma cells in the brain parenchyma preferentially involves vascular interactions.

Histological examination of the edges of the tumor masses from microcolonies in the spontaneous, intracardiac injection, and intraparenchymal models provided additional evidence for the predominant association of invasion with vessels. The leading edge from these tumors consisted of collective extensions/protrusions of tumor cells growing along adjacent vasculature (Figs. 1A, 1F, 3A, 3B, and S3; arrows). Collective angiotropic invasion was verified in the 4T1-GFP and B16F10-GFP models *in vivo* with transcranial imaging (Fig. 3D and S3). Human brain metastasis specimens were similarly examined for evidence of vascular associated parenchymal invasion. In 80% (8 of 10) cases of well-established solitary CNS breast cancer metastases, we could identify at least one area at the tumor-brain interface consistent with collective angiotropic invasion (Fig. 5D). Additionally, vascular association was observed in specimens showing single cell invasion (Fig. 5E). Finally, vascular-dependent invasion from the Virchow-Robin spaces was observed identically in both the mouse model and in carcinomatous human specimens (Fig. 5F). Therefore, metastatic carcinoma cells have limited ability to infiltrate neural substrates in vivo; instead, parenchymal invasion occurs predominantly via the vasculature consistent with an adhesive reliance on blood vessels.

### Adhesion to VBM promotes growth of metastatic cells

**Figure 6:**
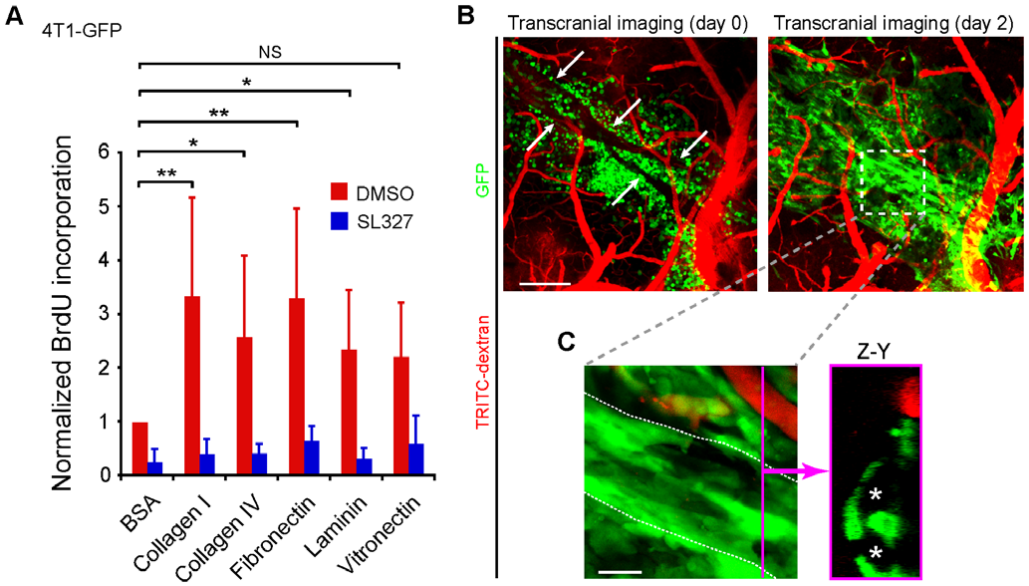
Brain vascular basement membrane potentiates carcinoma growth. (A) Basement membrane components (as indicated) potentiate incorporation of BrdU in 4T1-GFP cells in the absence of serum *in vitro* (*P<0.05, **P<0.001; ANOVA with Bonferroni Multiple Comparisons Test, experiment in triplicate, 3 repeats). BrdU incorporation was attenuated by treatment with the selective MEK inhibitor SL327 (all post-drug differences significant to *P*<0.01 except BSA). Error bars represent s.d. (B) After intraparenchymal injection of 4T1-GFP cells, several isolated meningeal vessels were placed over the injected tumor cells. The 4T1-GFP cells grew on the basement membrane of these non-perfused microvessels (outlined by arrows, left) *in vivo.* Growth was monitored with serial transcranial imaging; day 0 (left), day 2 (right). Scale bars, 240 µm. (C) Detail of hatched region in (B) demonstrating growth upon non-perfused vessels (hatched white lines). Reconstructed Z-Y dimension image at the level of the magenta line (right) demonstrates former lumens (asterisks) of the non-perfused vessels now surrounded by tumor cells. Scale bars, 30 µm (bottom).

The substrate for adhesion of carcinoma cells to brain vessels is likely the vascular basement membrane (VBM) which is composed predominantly of collagen type IV and laminins. Consistent with this hypothesis, the spatial distribution of collagen types I and IV, entactin fibronectin, laminin, and perlecan is limited to the exterior of blood vessels in the brain parenchyma (Fig. S6). These basement membrane proteins are known to promote anchorage-dependent growth signaling of adherent cells and are widely used to coat dishes for culture. To test the role of brain VBM in tumor cell growth signaling we examined whether adhesion to VBM components could potentiate metastatic cell proliferation *in vitro* in the absence of serum. 4T1-GFP cells were plated on collagen I, collagen IV, fibronectin, laminin, vitronectin, or BSA-coated control wells in the absence of serum. Cellular proliferation was measured at 48 h by BrdU incorporation ELISA. 4T1-GFP cells demonstrated significantly increased BrdU incorporation on all substrata compared to BSA (Fig. 6A). Consistent with the role of ERK in anchorage-dependent signaling for cellular survival and growth, this potentiation could be attenuated with incubation with the highly specific MEK inhibitor, SL327. Similar results were obtained with MDA-MB-231 and B16F10-GFP cells (Results not shown). Therefore, individual components of VBM are sufficient to support significant proliferation of metastatic tumor lines in the absence of serum *in vitro*.

We sought to verify that anchorage-dependent growth signaling from the VBM was a significant contribution to tumor growth *in vivo*. In order to distinguish use of the VBM from those growth signals and nutrients delivered directly from the bloodstream, we tested whether non-perfused vessels could act as sites for vascular cooption and tumor growth. Blood vessels were microdissected from the brain surface and placed upon the site of intraparenchmal tumor cell injection before implantation of a cranial window. Serial transcranial imaging was then performed to monitor tumor growth at 0, 2, and 4 days (Fig. 6B and results not shown). Widespread angiotropic growth and invasion occurred along the exterior of non-perfused vessels within just 2 d in 4 of 4 mice (Fig. 6C). This experiment suggests that adhesion to VBM of non-perfused vessels can promote vascular cooption and early growth of metastatic cells in the brain *in vivo*.

### The mechanism of vascular cooption in the CNS

We have shown that the vascular association of tumor cells in the CNS is based on adhesion to the VBM and that this relationship supports establishment and progression of tumor microcolonies independent of blood-born nutrients. Since cellular adhesion to vascular basement membrane components requires members of the integrin family of cellular adhesion receptors [29], it is likely that vascular cooption and subsequent metastatic tumor growth in the brain relies on identical mechanisms. As a corollary, we found marked immunoreactivity for activated focal adhesion kinase (pFAK-Y397), a major integrin signaling pathway, in 4T1-GFP microcolonies *in vivo* (Fig. S7). To test this hypothesis directly we investigated the effect of blocking integrin function on adhesion and metastatic colony growth. We focused this series of experiments on the human breast carcinoma line MDA-MB-231 because the integrin complement in this cell line has been well characterized [30] and due to the wider array of effective anti-integrin blocking antibodies available for human than for mouse. First, we confirmed expression of various integrin subunits on MDA-MB-231 microcolonies *in vivo* (Fig. S7) as previously described *in vitro* [30]. We next tested integrin subunit blocking antibodies on attachment to ECM in tissue culture. The anti-β1integrin subunit antibody alone nearly completely blocked adhesion of MDA-MB-231 cells to various types of ECM whereas antibodies to any other single α or β subunit did not similarly block adhesion (Fig. S7). Given the known functional redundancy in the complement of β1 integrins and the dominant role for β1 signaling in adhesion, migration and proliferation [31], this is not a surprising result. Indeed, all cell lines used in this study expressed high levels of β1 integrins (Fig. S7 and results not shown).

**Figure 7:**
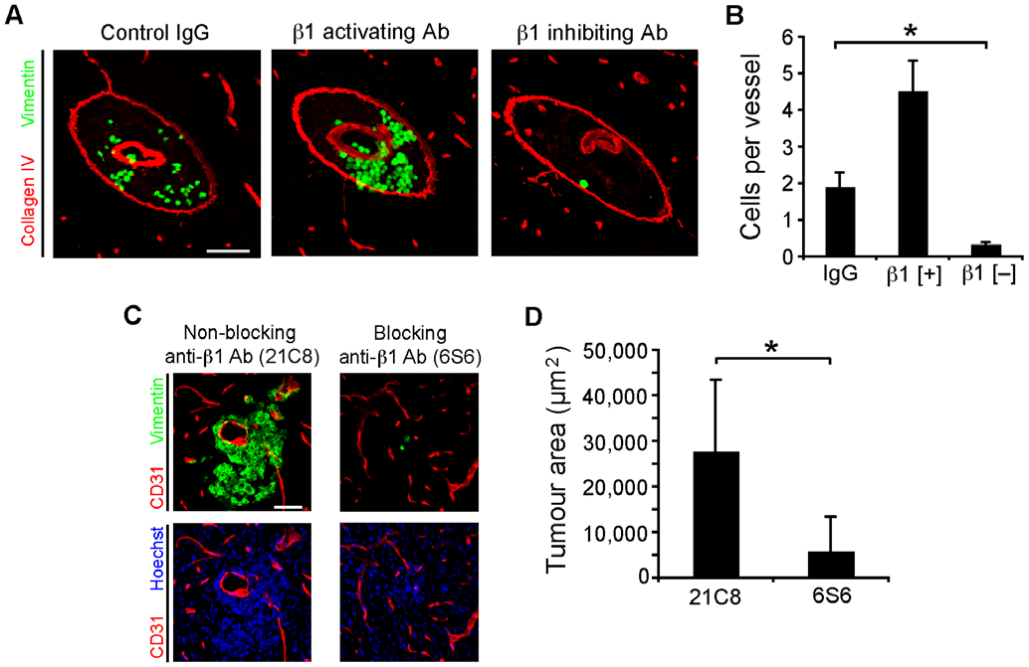
Adhesion to vascular basement membrane requires β1 integrin function. (A) MDA-MB-231 cells were tested for adhesion on slide-mounted human brain slices. Cells were plated in the presence of the indicated antibody and then washed after 2 h. Micrographs demonstrate adherent MDA-MB-231 cells (green) upon the same arteriole (red) in serial human brain sections under different treatment conditions (as indicated). Scale bar, 120 µm. (B) Adhesion to vessels was significantly attenuated with anti-β1 function blocking antibodies (*P<0.001, Kruskal-Wallis test with post-hoc Dunn's multiple comparisons test, tissue from 2 patients run in duplicate and repeated) and potentiated with an activating anti-β1 antibody. (C) MDA-MB-231 cells (vimentin+, green) were treated with β1 integrin subunit blocking or non-blocking antibodies prior to intraparenchymal injection in the brain of SCID mice. After 4 d, tumor colonies from animals in the non-blocking antibody condition (left panels) demonstrated vascular associated growth and invasion in contrast to the blocking condition (right panels). Horizontal sections. Scale bar, 60 µm. (D) Tumor cross sectional area was measured after 4 d growth with antibody treated cells showing significantly larger tumor area in the non-blocking condition (*P<0.05, Mann-Whitney U-test, *n* = 3–4 mice per treatment).

To test the importance of β1 integrins in brain metastasis, we assayed tumor cell adhesion on a more physiologically-relevant substrate using brain sections. MDA-MB-231 cells were preincubated with either isotype-matched IgG1 control or anti-β1 activating or inhibiting antibodies prior to plating on adjacent unfixed slide-mounted human brain sections (Fig. 7A). The tumor cells when plated on brain sections had been noted to adhere almost exclusively to vessels (Fig. 4C and 4D). Adhesion to vessels was potentiated by anti-β1 integrin activating antibodies and markedly attenuated by anti-β1 integrin blocking antibody (Fig. 7A and 7B). Thus, adhesion of tumor cells to the VBM, and consequently the process of vascular cooption, depends upon tumor cell β1 integrins.

To investigate the relevance of the above findings *in vivo* we asked whether blocking the β1 integrin subunit would affect colony formation after intraparenchymal injection of tumor cells. MDA-MB-231 cells were incubated with monoclonal antibodies which block the function of the human, but not murine, β1 integrin subunit (Results not shown). Axial tumor area was evaluated 4 d after the injection on histopathology (Fig. 7C). As predicted, the brain colonies derived from cells treated with non-blocking antibodies were significantly larger than those from the cells treated with blocking antibodies (Fig. 7D). We verified the antibody treatment itself was not toxic to these tumor cells after culture in the absence of serum and ECM substrate (Results not shown). Thus, cells treated with β1 integrin subunit blocking antibodies largely failed to become established and grow in the brain consistent with the inability to adhere to and utilize the surrounding blood vessels.

### Brain metastasis establishment requires β1 integrin-mediated proliferation

**Figure 8:**
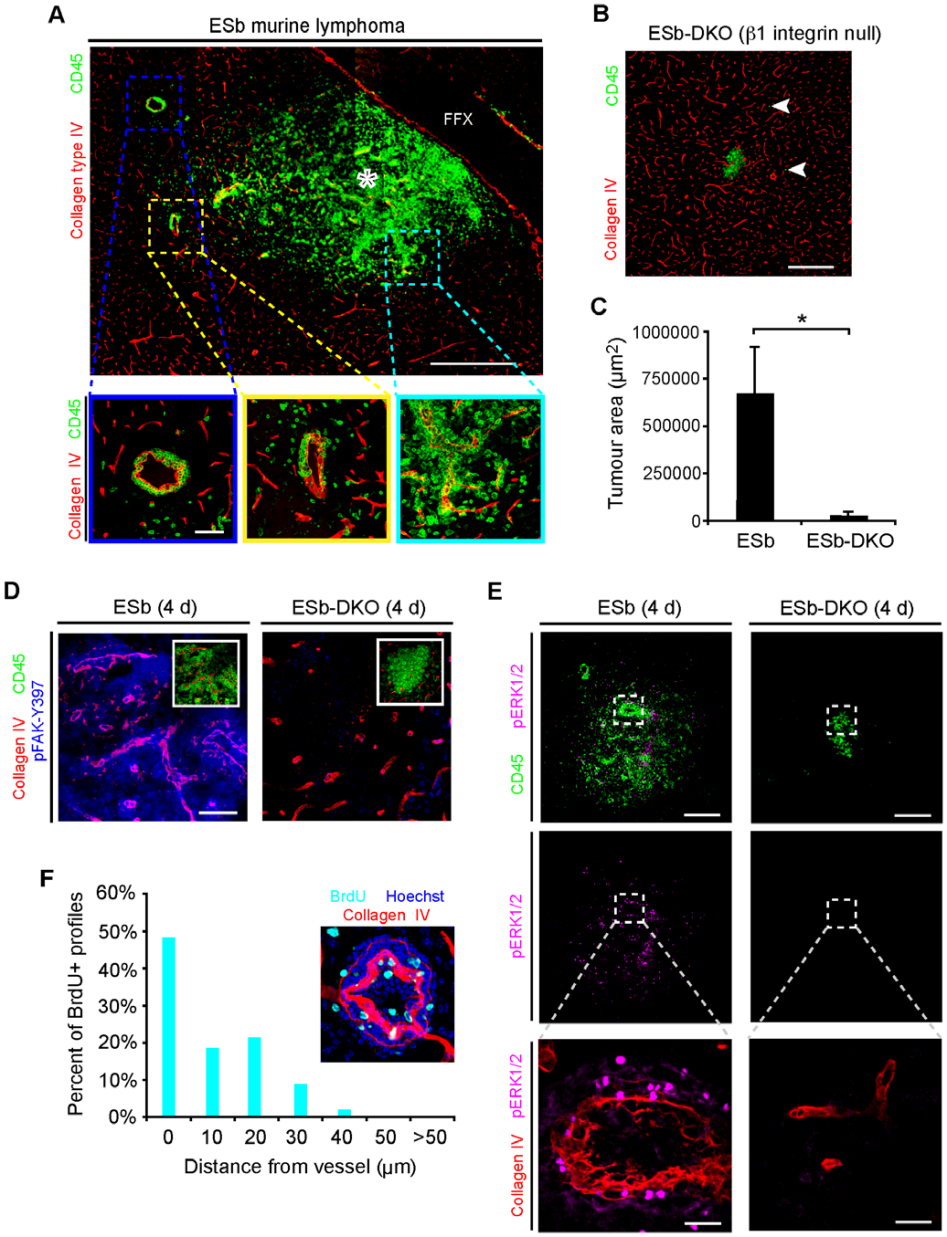
β1 integrin is required for angiocentric growth of tumor cells in the CNS *in vivo*. (A) Representative montage demonstrates tumor growth and invasion of ESb cells (CD45+, green) from the point of initial injection (asterisk). Despite the ability of single cells to migrate through the brain parenchyma there was a clear preference for growth upon vessels (hatched areas, lower panels). Inset, lower left demonstrates BrdU+ (cyan) perivascular ESb cells. Scale bar, 500 µm (montage), 60 µm (bottom micrographs). FFX, fimbria/fornix bundle. (B) *in vivo* growth of ESb-DKO murine lymphoma cells (β1 integrin null) is highly impaired relative to ESb cells (A). Long-distance migration of single cells is not affected (arrowheads). Scale bar, 240 µm. (C) Tumor area was nearly 22 times greater in the ESb compared to the ESb-DKO line at 4 d (*p<0.01; error bars represent s.d.; *n* = 3–4). (D) Immunohistochemistry for activated FAK (pFAK-Y397) demonstrates strong staining in perivascular wild-type ESb cells (left) but little staining in ESb-DKO cells (right). Scale bar, 60 µm. Asterisk, injection site. (E) Immunohistochemistry for activated ERK (pERK1/2) shows strong nuclear staining and moderate cytoplasmic staining of perivascular ESb lymphoma cells (left) but not in ESb-DKO cells (right). Scale bars, 240 µm (top) and 30 µm (bottom). (F) Histogram demonstrating perivascular distribution of BrdU+ profiles in animals injected with ESb cells (cyan). The majority of BrdU+ profiles are within 1 cell diameter of vessels (mean ± s.d. distance: 7.5±9.4 µm).

The above results with function blocking antibodies identify an important role for β1 integrins in metastatic tumor microcolony establishment in the CNS. To further evaluate the effect of β1 integrins on colony formation, we analyzed CNS growth of ESb murine lymphoma cells and of an engineered mutant in which the β1 integrin subunit has been genetically eliminated (ESb-DKO) [32]. Both ESb and ESb-DKO cells proliferate normally in suspension *in vitro* under standard defined medium conditions ([32] and Results not shown) and are not susceptible to anoikis. Similar to the pathology seen in many human lymphomas, and in contrast to that of carcinoma metastases, ESb cells diffusely invaded through the parenchyma, but demonstrated superimposed perivascular cuffing reminiscent of cooption (Fig. 8A). These cells formed a rapidly growing lesion often measuring over a millimeter in diameter in just 4 d. In contrast, the β1 integrin subunit deficient ESb-DKO cells were significantly impaired in the ability to grow and form an expanding lesion in the brain (Fig. 8B and 8C). ESb-DKO cells retained the capability for invasion deep into the brain parenchyma confirming that the β1 integrin subunit is not necessary for lymphoma invasion of the CNS [33] (Fig. 8B, arrows). We noted a low baseline of BrdU+ profiles at the injection site of mice and widespread invasion and BrdU-positive cells throughout the cranial fascia and dermis overlying the injection site in mice injected with ESb-DKO ruling out a proliferative defect in the mutant line *in vivo* (Fig. S8) [32].

To verify that ESb cells interacted with the VBM *in vivo*, we analyzed tissue sections with immunohistochemistry against activated focal adhesion kinase (pFAK-Y397) and activated extracellular regulated kinase (pERK1/2). ESb cells were highly immunoreactive for pFAK-Y397 confirming integrin-mediated anchorage-dependent signaling (Fig. 8D). In contrast, ESb-DKO cells demonstrated only background levels of immunoreactivity for pFAK-Y397. ERK activity is downstream of FAK activation and associated with survival and mitosis. We observed strong nuclear and moderate cytoplasmic pERK1/2 immunoreactive ESb lymphoma cells, but only weakly positive ESb-DKO cells in brain colonies (Fig. 8E). In addition, ESb-colonized vessels demonstrated frequent perivascular BrdU positive nuclei (Fig. 8F, inset). Interestingly the majority of BrdU+ cells (67%) were found in direct contact or within 10 µm of vessels (Fig. 8E). Therefore, ESb, but not ESb-DKO, tumor cells in the brain were associated with immunoreactivity for activated FAK and ERK as well as perivascular BrdU consistent with proliferation via anchorage dependent signaling from the VBM. Thus, the β1 integrin subunit on tumor cells mediates anchorage-dependent growth signaling upon adhesion to the VBM of the neurovasculature and is essential for the formation of metastatic brain colonies by ESb cells. Further, the requirement for the β1 integrin subunit is independent from protection from anoikis in this case.

## Discussion

We have demonstrated that the primary soil for metastatic tumor cell attachment and growth in the brain is vascular rather than neural. This vascular cooption, or the utilization of pre-existing vessels, has only been previously anecdotally reported as a form of vascularization in experimental brain metastasis (Table S1). Here, we have quantitatively demonstrated it to be the predominant form of vessel use by tumor cells during early experimental brain metastasis establishment and in human clinical specimens reflecting early stages of the disease. We further show the interaction relies on β1 integrin-mediated tumor cell adhesion to the vascular basement membrane of blood vessels. These findings exclude a requirement for *de novo* angiogenesis prior to microcolony formation. They also contrast with the classical seed and soil hypothesis for brain metastasis suggesting a neural substrate and reliance upon neural-derived trophic factors for growth. Importantly, they do not exclude vascular remodeling [24] or contributions from the neural elements for later growth [34]. This work thus describes in detail a major mechanism of brain metastasis formation in addition to identifying the mechanism of vessel cooption in the brain for the first time.

The CNS parenchyma is largely devoid of non-vascular stromal basement membrane components which are necessary for epithelial and carcinoma cell adhesion and survival (Fig. S6). Vascular cooption, therefore, supplies substrates for malignant growth of non-neural carcinoma cells not otherwise widely available in the neuropil. Proliferation by metastatic tumor cells is highly potentiated upon adhesion to a basement membrane substratum and is attenuated by inhibiting MEK *in vitro*. Consistent with the experiments in tissue culture, during the early stages of colony formation *in vivo* we found the vast majority of micrometastases to be in direct contact with the VBM of existing brain vessels and many of these cells were proliferating.

Resident neural stem cells tend to localize in perivascular locations [35] and cells defined as brain tumor stem cells (BTSCs) are found in a similar location [36], [37]. Secreted paracrine growth factors from the endothelial cells of the “perivascular niche” were shown to stimulate the growth and survival of BTSCs. In contrast, we demonstrated that slide-mounted brain sections (i.e., dead tissue) still supplied the components needed for adhesion and spreading by carcinoma cells. The requirement of metastatic carcinoma cells for the vasculature in adhesion and invasion during metastasis in the brain may be more analogous to the requirement for VBM during development of pancreatic islets [38]. Islet cells use β1 integrins to interface with the VBM and this interaction is required for proliferation and endocrine function. Nikolova et al. termed this basement membrane microenvironment, a “vascular niche” [38], [39]. Similarly vascular mural cells require the β1 integrin subunit for proper adhesion to vessels and for maintaining vessel stability [40]. In an analogous fashion, carcinoma cells, then, appear to hijack the brain's VBM for essential functions during brain metastasis. Interestingly, inhibiting angiogenesis in circumscribed, well-established CNS melanoma metastases causes reversion to growth by vascular cooption [19]. This suggests a continuum for vessel utilization by tumor cells which may represent a viable target for therapeutic exploitation.

The interaction between the tumor cells and the vessels relies on β1 integrin-mediated tumor cell adhesion to the vascular basement membrane of blood vessels. This interaction is sufficient to promote immediate proliferation and micrometastasis establishment of tumor lines in the brain. This angiotropic mechanism was universal to both carcinomas (anchorage-dependent cells) and lymphomas (anchorage-dispensible cells) in the CNS. β1 integrins play a dominant role in many facets of normal cell biology and have been implicated in cancer initiation, progression, and metastasis [30]–[32], [41]–[44]. There are at least 10 β1 integrin heterodimers which serve as variably promiscuous adhesive receptors to diverse ligands such as the collagens and laminins. Nonetheless our data suggest that antagonism of the β1 integrin subunit alone might be useful in therapeutic strategies for brain metastases. Indeed, Park et al [42] found that inhibitory anti-β1 integrin subunit antibodies induced apoptosis in breast carcinoma cells grown in three dimensional culture, but not in cells grown in monolayers. Treating mice bearing breast cancer xenografts from those cell lines with the same antibody led to decreased tumor volume. In addition to the apoptotic mechanism described *in vitro*, inhibition of vascular cooption may have also attenuated growth. In an alternative strategy to evaluate the role of β1 integrins, tumors were analyzed in the MMTV/PyMT transgenic model of breast cancer [43]. Conditional deletion of β1 integrin after induction of tumorigenesis resulted in impairment of FAK phosphorylation and proliferation consistent with a reliance on anchorage-dependent signaling for tumor growth. Although the presence of the β1 integrin subunit is mandatory for embryonic development, chronic systemic anti-β1 antibody therapy did not result in overt toxicities in adult mice [42]. Further research into the regulation of β1 integrin regulation, function and downstream signaling may yield clinically useful applications for metastatic disease in cancer patients [31], [45], [46].

## Materials and Methods

### Ethics statement

Animal procedures were performed under UK Home Office licensing and ethics committee approval from the Clinical Medicine Ethical Review Committee at the University of Oxford. Human tissue was retrieved from the Thomas Willis Oxford Brain Collection with prior written informed consent and final approval from the local research ethics committee (reference 06/Q1604/141).

### *In vivo* experiments

All animal procedures were approved by the UK Home Office. Experimental brain metastases were established by intracardiac injection of 10^5^ tumor cells [11]. Alternatively, direct intraparenchymal injection of 5×10^3^ to 10^4^ cells was performed into the striatum or hippocampus with a stereotaxic apparatus (Benchmark, MyNeurolab.com). MRI imaging was performed with a 7-Tesla horizontal bore magnet with a Varian Inova spectrometer (Varian) between 2 and 14 d post tumor inoculation. Transcranial imaging was performed up to 3 times via cranial window with a Leica TCS-SP2 AOBS confocal microscope and a Spectra-Physics MaiTai Ti-Sapphire pulsed laser.

### *In vitro* experiments

VBM adhesion and proliferation assays were performed with 10 µg/ml ECM components or BSA (control). Adhesion was tested after 2 h and proliferation after 48 h. Adhesion to brain sections was evaluated after 2 h [28]. Non-adherent cells were rinsed off in three rinses of PBS on a shaker set. Live brain slice assays were performed acutely for 2 hours or 3 to 7 d for long-term culture as described in Text S1.

### Human specimens

Clinical neuropathology specimens were obtained with ethics committee approval from routine neurosurgical or autopsy procedures and processed for routine immunohistochemistry and analyzed as described in Text S1.

### Integrin inhibition studies

Inhibitory monoclonal antibodies against integrin subunits and isotype control antibodies were used at 5 to 20 µg/ml.

### Histological analysis

Experimental tissues were collected under terminal anesthesia after transcardiac perfusion with saline and 4% paraformaldehyde or organs were freshly isolated and snap frozen or immersion fixed. Immunohistochemistry was performed as described in Text S1 in 15-30 µm cryostat sections.

### Statistical analyses

Data were compiled in MS Excel and data analyzed with Excel or Graph Pad InStat 3. Pairwise comparisons were made with an unpaired t-test or the Mann-Whitney u-test as appropriate. Evaluation of variance in data groups was performed with ANOVA or the Kruskal-Wallis test as appropriate. If significance was detected post-hoc comparisons were made with an appropriate post-hoc test. A *p*<0.05 was considered statistically significant.

For detailed methodology please see Text S1.

## Supporting Information

Table S1 (0.05 MB DOC)

Figure S1 Brain seeking MDA231BR line requires vascular cooption for CNS growth. (A) Intracardiac injection of metastatic human breast carcinoma MDA-MB-231 or the brain seeking subclone, MDA231BR, results in tumour growth on existing brain vessels at 7–14 d. Vascular association of colonies for either cell line was>97% (left) (B) despite a greater number of brain colonies formed after injection of MDA231BR than after the parental line (middle, *P<0.05, Mann-Whitney U-test). (C) Tumour area from each subclone was also equivalent (right). These results further support the hypothesis that interactions with existing vessels are necessary for initial growth of brain metastases. All error bars represent s.d. (n = 3–4 mice per cell line).
(0.06 MB TIF)

Figure S2 Experimental brain micrometastases coopt and grow upon pre-existing vessels. (A) Representative images of tumor-associated cortical vessels or control hemisphere visualized by Glut-1 immunoreactivity (red), a biomarker for an intact blood-brain barrier (BBB). Inset shows the vascular associated tumor cells (green) superimposed on the vasculature. Scale bar, 120 µm. (B) Quantitation demonstrates significantly lower vascular density in regions with growing brain metastases compared to corresponding fields in control brains. (*P<0.05, t-test; n = 3 per group). Error bars represent s.d. (C) High resolution T2-weighted and gadolinium-dTPA enhanced T1-weighted MRI largely failed to reveal experimental brain microcolonies at timepoints between 7 and 14 d after intracardiac inoculation (n = 5). This is consistent with the lack of blood brain barrier (BBB) leakage as would be expected from new tumour vessels. Yellow arrowhead, high intensity signal in sagittal sinus serves as positive control for gadolinium enhancement. Bottom, representative brain section (fluorescent montage) at +4.0 Bregma demonstrates numerous tumour microcolonies (white arrowheads) which were not detected by MRI. Scale bar, 1 mm (montage). (D) BBB integrity was further verified with enzymatic immunofluorescence for mouse IgG on adjacent sections. Middle, high power micrograph of boxed area in (C) displays a 4T1-GFP microcolony with no detectible frank BBB disruption. Positive and negative controls as indicated. High concentration of IgG in microglia and vessels as previously described [47]. Arrows, microglia; arrowheads, vessels. Scale bar, 40 µm (micrograph).
(1.75 MB TIF)

Figure S3 Active vascular preference of carcinoma cells in the brain in vivo. (A) 1 h after intraparenchymal injection of 4T1-GFP cells into BALB/c mice, cells were visualized through a cranial window. Tumor cells could be seen spreading along the pre-existing vessels (arrow). Scale bar, 15 µm. (B) B16F10-GFP murine metastatic melanoma cells associate with preexisting vessels in the CNS after intraparenchymal injection. Left, histological section at 4 d. Right, imaging vascular invasive cells through cranial window in a live anesthetized mouse. Arrows, angiocentric invasion. Scale bars, 30 µm.
(0.86 MB TIF)

Figure S4 Carcinoma cell spreading on vessels in live brain slices. (A) Distribution of cell morphologies after co-culture with acutely isolated living brain slices. 5×103 tumour cells were plated on each brain slice and analysed for morphology after 2 hours. Elongated cells represented a small subset of cells in all tumour lines. (B) All cells were scored in regard to contact with blood vessels and graphed according to morphology. Indeed, upwards of 90% of elongated cells for all 5 cell lines were in contact with blood vessels. There were significantly more vascular associated elongated cells compared to round cells associated with vessels (p<0.01 for all cell lines, Kruskal-Wallis test with post-hoc Dunn's multiple comparisons test, error bars represent s.d.). This suggests vascular contact is causal in the ability for the cells to spread out or elongate on brain slices. (C-F), Representative fields of the various cell lines (as indicated) plated upon live brain slices demonstrating vascular preference of elongated cells. Right panels (C-F) represent high power views of hatched areas for greater detail. Arrows, elongated vascular associated cells. MDA-MB-231, MDA231BR, and A7 cells are identified by vital staining with CMRA prior to co-culture (red). Scale bars, 120 µm (C, D, and F), 60 µm (E).
(0.87 MB TIF)

Figure S5 Carcinoma cells preferentially adhere to brain vessels in situ. (A) Adherent MDA-MB-231 cells appeared to prefer cross-sectional arteries and arterioles as a substrate (see Fig. 4D) in human tissue and were found to adhere especially to the muscular layer of the vessel wall. This layer, found between the media intima and adventitia, possesses a fine reticular meshwork of vascular basement membrane proteins which likely serves as the primary adhesion substrate (right panels; scale bars, 60 µm, left; 15 µm, right.). The seeming arterial preference may be due to the larger exposed area of basement membrane of arterioles compared to (B) veins and (C) capillaries. White arrow, media intima; yellow arrow, media adventitia. Scale bars (B and C), 120 µm.
(2.17 MB TIF)

Figure S6 Basement membrane proteins are limited to brain blood vessels (A) Distribution of collagen type I, collagen type IV, entactin, fibronectin, laminin, and perlecan in the normal murine brain was evaluated with immunofluorescence on horizontal sections. Micrographs were acquired in the cortex. The VBM components were largely limited to the vasculature as demonstrated by co-localisation with the endothelial cell markers Glut-1 or CD34 (green). White arrowhead, collagen type I expression in pia mater. Scale bar, 60 µm. (B) In contrast, immunostaining of normal mouse visceral organs (as indicated) produced vascular and extensive extravascular immunoreactivity for laminin. Scale bar, 60 µm.
(2.84 MB TIF)

Figure S7 Integrins mediate adhesion to brain vessels. (A) Murine mammary carcinoma 4T1-GFP brain microcolonies are immunoreactive to activated focal adhesion kinase (pFAK-Y397) suggestive of integrin-mediated downstream signaling. Scale bars, 60 µm (left) and 30 µm (right). (B) Expression of integrin subunits previously reported to be expressed in MDA-MB-231 cells in vitro [30] was verified in experimental brain metastases in vivo 7 d after intracardiac injection. All eight subunits (α6, not shown) were readily detectible in microcolonies with indirect immunofluorescence using human-specific monoclonal antibodies. Scale bar, 15 µm. (C) MDA-MB-231 breast carcinoma cells were tested for adhesion to human placental collagen type IV or laminin in the presence of the indicated blocking antibodies (* p>0.01, ANOVA with post-hoc Dunnet multiple comparisons test, performed in quadruplicate and repeated). (D) The effect of blocking antibodies to human β1 or β3 integrin subunits, of control IgG, or of activating β1 integrin subunit antibody was tested on adhesion of MDA-MB-231 human breast carcinoma cells to mouse EHS collagen type IV and laminin (p>*0.01 both comparisons, ANOVA with post-hoc Dunnet multiple comparisons test, performed in triplicate or quadruplicate and repeated). These experiments identified the β1 subunit to be obligatory for MDA-MB-231 breast carcinoma adhesion to murine EHS collagen type IV and laminin and human HP collagen type IV. (E) Western blot for the carboxy terminus of the β1 integrin subunit reveals expression in all tumor cell lines used in this study analyzed (other lines not shown). Numerous glycosylated isoforms [45] as well as species-specific variants can be appreciated. β actin served as loading control. (F) β1 integrin subunit immunofluorescence on the cell membrane in both 4T1-GFP and MDA-MB-231 (as indicated) cells plated in collagen type IV coated wells. Immunoreactivity was also seen particularly distally in cell processes of MDA-MB-231 cells (arrows). Scale bar, 30 µm (top), 10 µm (bottom).
(0.67 MB TIF)

Figure S8 β1 integrin null ESb-DKO cells are not generally growth defective in vivo. (A) BrdU immunohistochemistry (cyan) of tissue sections from DBA/2 mice 4 d after intraparenchymal injection of ESb-DKO cells (green, inset) demonstrates a low baseline of proliferation (horizontal brain section). Scale bar, 30 µm. (B) ESb-DKO cells are able to invade, grow, and proliferate (inset) within several layers of the scalp over the injection site (arrow, hair follicle; coronal brain section). Scale bar, 240 µm.
(0.43 MB TIF)

Text S1 Detailed Experimental Procedures
(0.14 MB DOC)

Movie S1 Tumor cell spreading on live brain slices requires vascular contact. Timelapse confocal microscopy of A7 human malignant melanoma cells (red) plated onto a live SCID brain slice demonstrates a vascular associated cell elongating (center) upon a large exposed vessel (green) over the course of 30 minutes. Vasculature vitally stained with GS-IB4 lectin (Alexa 488). Z-stacks were captured every 3 m and 2D z-projections compiled with ImageJ.
(0.05 MB MOV)
