# Supplementary material for: The Vascular Basement Membrane as “Soil” in Brain Metastasis
Source: PLoS One. 2009 Jun 10;4(6):e5857. doi: 10.1371/journal.pone.0005857 (PMC2689678; doi:10.1371/journal.pone.0005857)
Supplement: Table S1 — (0.05 MB DOC) [file pone.0005857.s001.doc]

**Table S1.** Experimental brain metastases are highly associated with pre-existing vessels.

| Ref. | Cell Lines | Host | Model System | Example |
| --- | --- | --- | --- | --- |
| [15] | B16-B10n (murine melanoma) | Syngeneic C57BL/6 mice | Intravenous injection (tail vein) of 2- to 5x10^4^ cells | Fig. 1 |
| [16] | MDA231BR (human breast carcinoma) | BALB/c nude or NIH *nu/nu* mice | Intracardiac injection of 1.0x10^4^ to 1.75x10^5^ cells | Figs. 1A, 1C |
| [17] | Mel57, M14, & 530 (human melanoma) | BALB/c *nu/nu* mice | Intracarotid injection | Figs. 1A, 1E, and 1F |
| [18] | Mel57-VEGF-A | BALB/c *nu/nu* mice | Intracarotid injection | Fig. 4E |
| [19] | 4T1 & 6T2 (melanoma-lymphocyte hybrids) | P1-P4 C57BL/6 mice | Intraparenchymal injection of 1x10^5^ cells | Figs. 3, 5 |
| [20] | B16-B14b (murine melanoma) | Syngeneic C56BL/6 mice | Intracarotid injection of 1x10^5^ cells | Fig. 4 |
| [21] | UV-2237 MM (murine fibrosarcoma) | Syngeneic C3H/HeN mice | Intracarotid injection of 1x10^5^ cells | Figs. 2A, 2B |
