# Supplementary figures and images for: The Vascular Basement Membrane as “Soil” in Brain Metastasis
Source: PLoS One. 2009 Jun 10;4(6):e5857. doi: 10.1371/journal.pone.0005857 (PMC2689678; doi:10.1371/journal.pone.0005857)

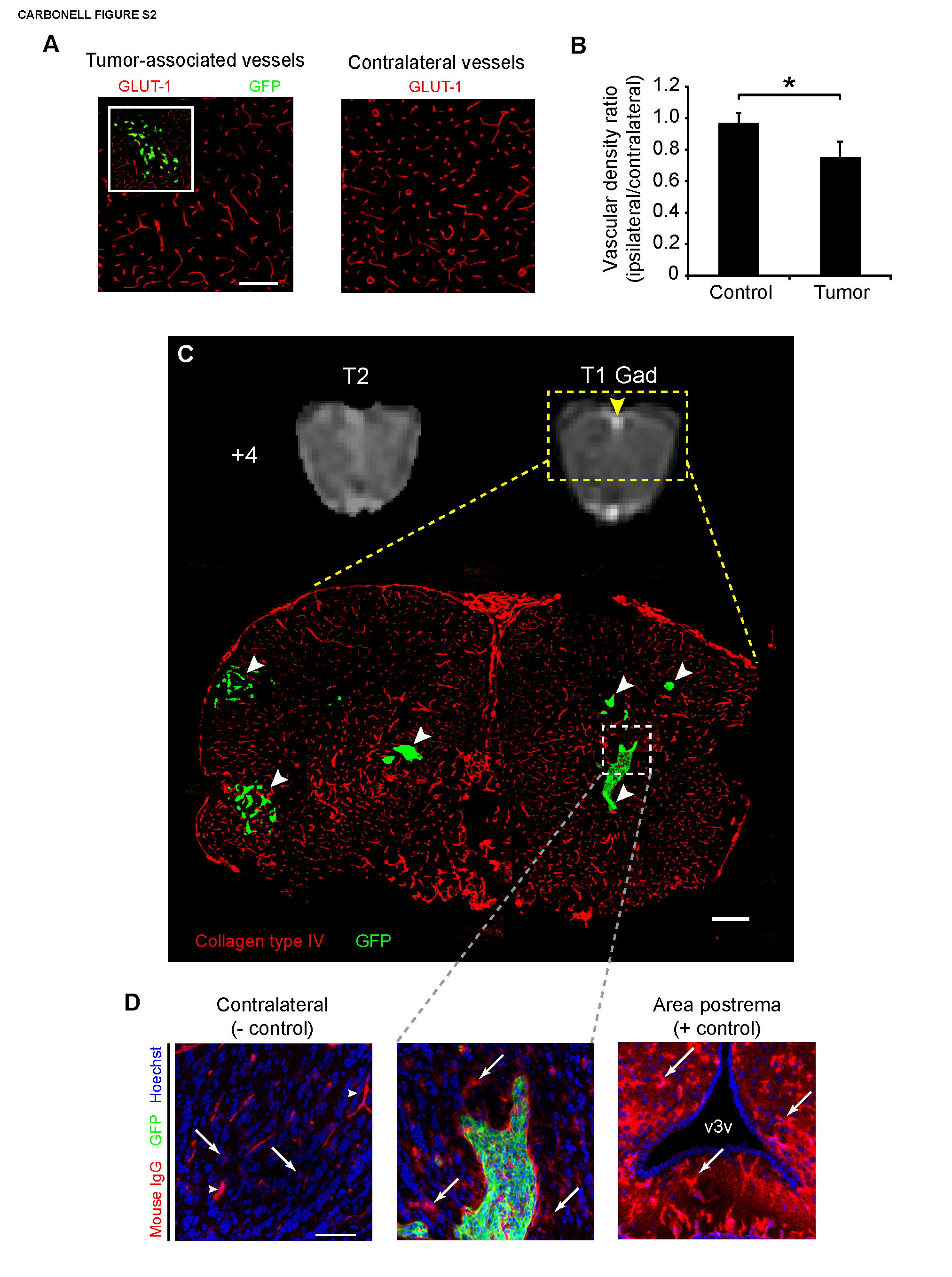

Supplement: Figure S2 — Experimental brain micrometastases coopt and grow upon pre-existing vessels. (A) Representative images of tumor-associated cortical vessels or control hemisphere visualized by Glut-1 immunoreactivity (red), a biomarker for an intact blood-brain barrier (BBB). Inset shows the vascular associated tumor cells (green) superimposed on the vasculature. Scale bar, 120 µm. (B) Quantitation demonstrates significantly lower vascular density in regions with growing brain metastases compared to corresponding fields in control brains. (*P<0.05, t-test; n = 3 per group). Error bars represent s.d. (C) High resolution T2-weighted and gadolinium-dTPA enhanced T1-weighted MRI largely failed to reveal experimental brain microcolonies at timepoints between 7 and 14 d after intracardiac inoculation (n = 5). This is consistent with the lack of blood brain barrier (BBB) leakage as would be expected from new tumour vessels. Yellow arrowhead, high intensity signal in sagittal sinus serves as positive control for gadolinium enhancement. Bottom, representative brain section (fluorescent montage) at +4.0 Bregma demonstrates numerous tumour microcolonies (white arrowheads) which were not detected by MRI. Scale bar, 1 mm (montage). (D) BBB integrity was further verified with enzymatic immunofluorescence for mouse IgG on adjacent sections. Middle, high power micrograph of boxed area in (C) displays a 4T1-GFP microcolony with no detectible frank BBB disruption. Positive and negative controls as indicated. High concentration of IgG in microglia and vessels as previously described [47]. Arrows, microglia; arrowheads, vessels. Scale bar, 40 µm (micrograph). (1.75 MB TIF) [file pone.0005857.s003.tif]

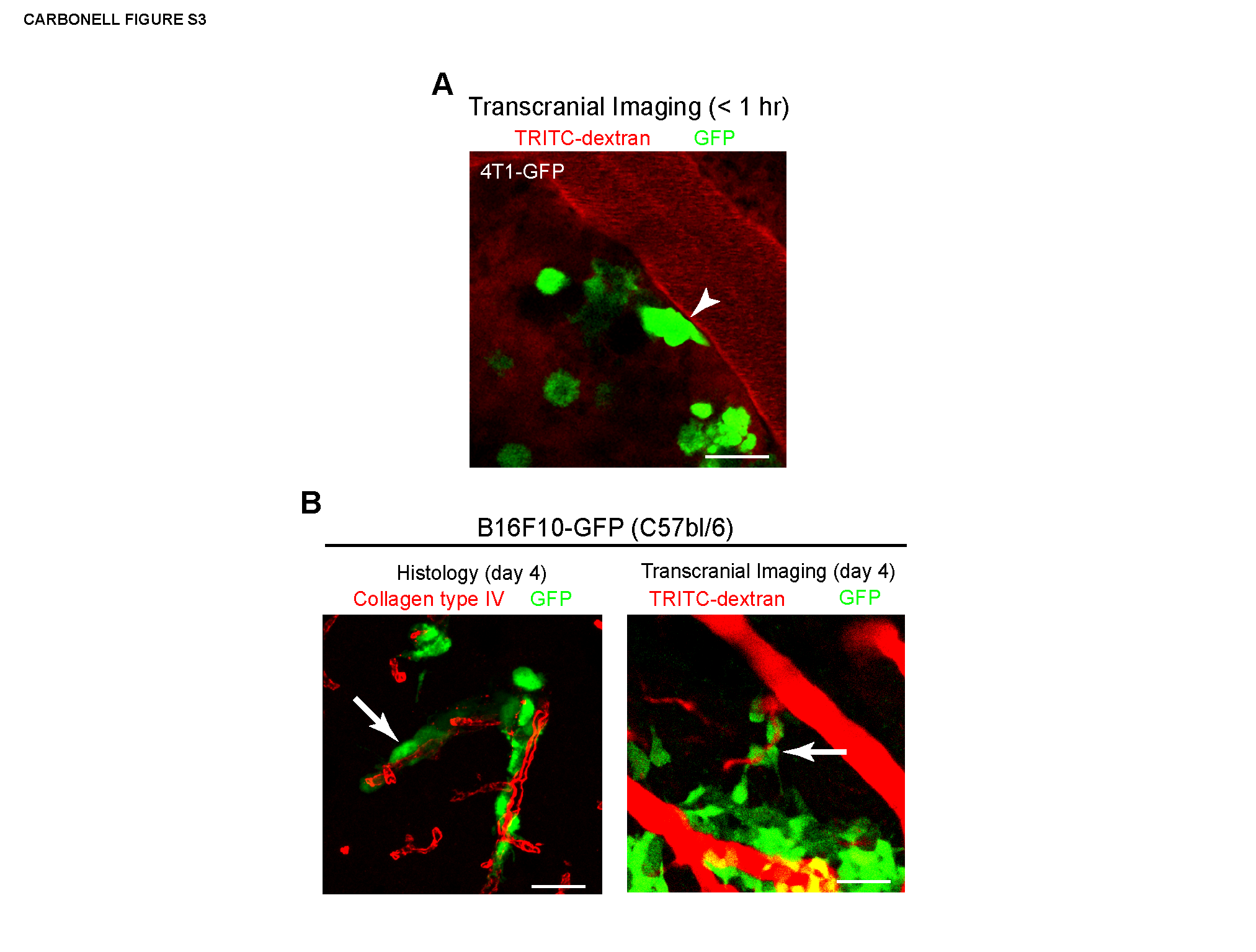

Supplement: Figure S3 — Active vascular preference of carcinoma cells in the brain in vivo. (A) 1 h after intraparenchymal injection of 4T1-GFP cells into BALB/c mice, cells were visualized through a cranial window. Tumor cells could be seen spreading along the pre-existing vessels (arrow). Scale bar, 15 µm. (B) B16F10-GFP murine metastatic melanoma cells associate with preexisting vessels in the CNS after intraparenchymal injection. Left, histological section at 4 d. Right, imaging vascular invasive cells through cranial window in a live anesthetized mouse. Arrows, angiocentric invasion. Scale bars, 30 µm. (0.86 MB TIF) [file pone.0005857.s004.tif]

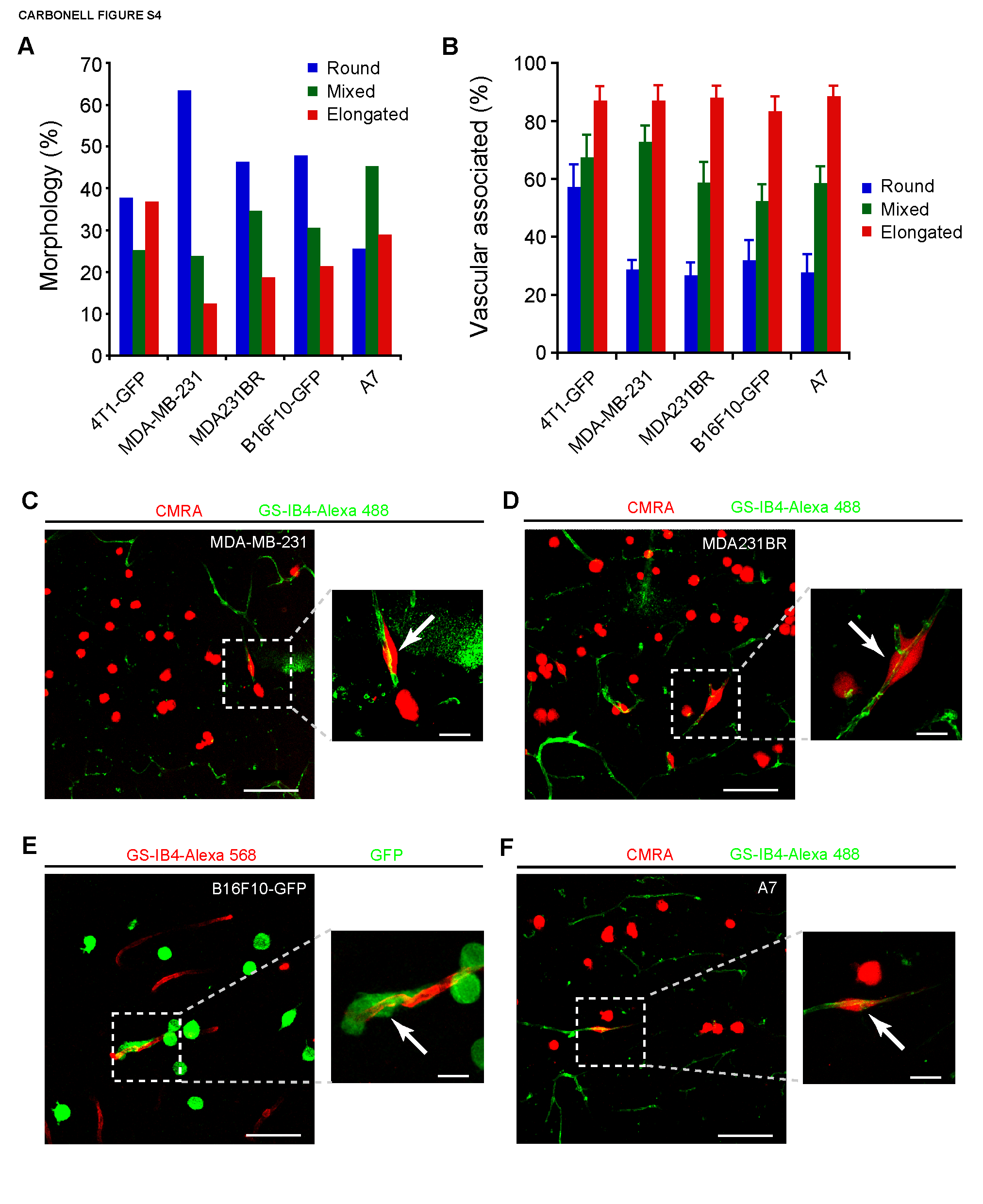

Supplement: Figure S4 — Carcinoma cell spreading on vessels in live brain slices. (A) Distribution of cell morphologies after co-culture with acutely isolated living brain slices. 5×10^3^ tumour cells were plated on each brain slice and analysed for morphology after 2 hours. Elongated cells represented a small subset of cells in all tumour lines. (B) All cells were scored in regard to contact with blood vessels and graphed according to morphology. Indeed, upwards of 90% of elongated cells for all 5 cell lines were in contact with blood vessels. There were significantly more vascular associated elongated cells compared to round cells associated with vessels (p<0.01 for all cell lines, Kruskal-Wallis test with post-hoc Dunn's multiple comparisons test, error bars represent s.d.). This suggests vascular contact is causal in the ability for the cells to spread out or elongate on brain slices. (C-F), Representative fields of the various cell lines (as indicated) plated upon live brain slices demonstrating vascular preference of elongated cells. Right panels (C-F) represent high power views of hatched areas for greater detail. Arrows, elongated vascular associated cells. MDA-MB-231, MDA231BR, and A7 cells are identified by vital staining with CMRA prior to co-culture (red). Scale bars, 120 µm (C, D, and F), 60 µm (E). (0.87 MB TIF) [file pone.0005857.s005.tif]

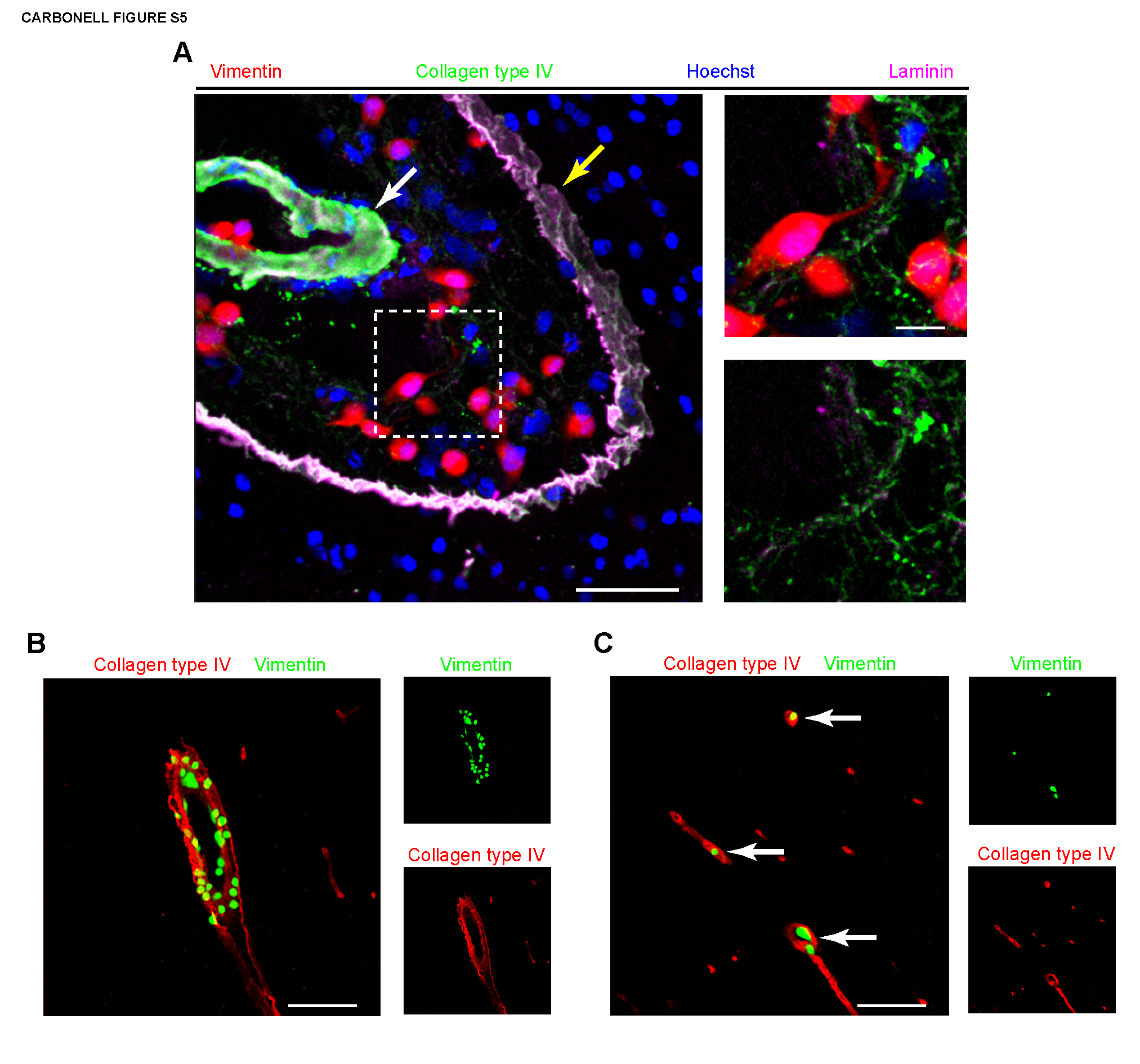

Supplement: Figure S5 — Carcinoma cells preferentially adhere to brain vessels in situ. (A) Adherent MDA-MB-231 cells appeared to prefer cross-sectional arteries and arterioles as a substrate (see Fig. 4D) in human tissue and were found to adhere especially to the muscular layer of the vessel wall. This layer, found between the media intima and adventitia, possesses a fine reticular meshwork of vascular basement membrane proteins which likely serves as the primary adhesion substrate (right panels; scale bars, 60 µm, left; 15 µm, right.). The seeming arterial preference may be due to the larger exposed area of basement membrane of arterioles compared to (B) veins and (C) capillaries. White arrow, media intima; yellow arrow, media adventitia. Scale bars (B and C), 120 µm. (2.17 MB TIF) [file pone.0005857.s006.tif]

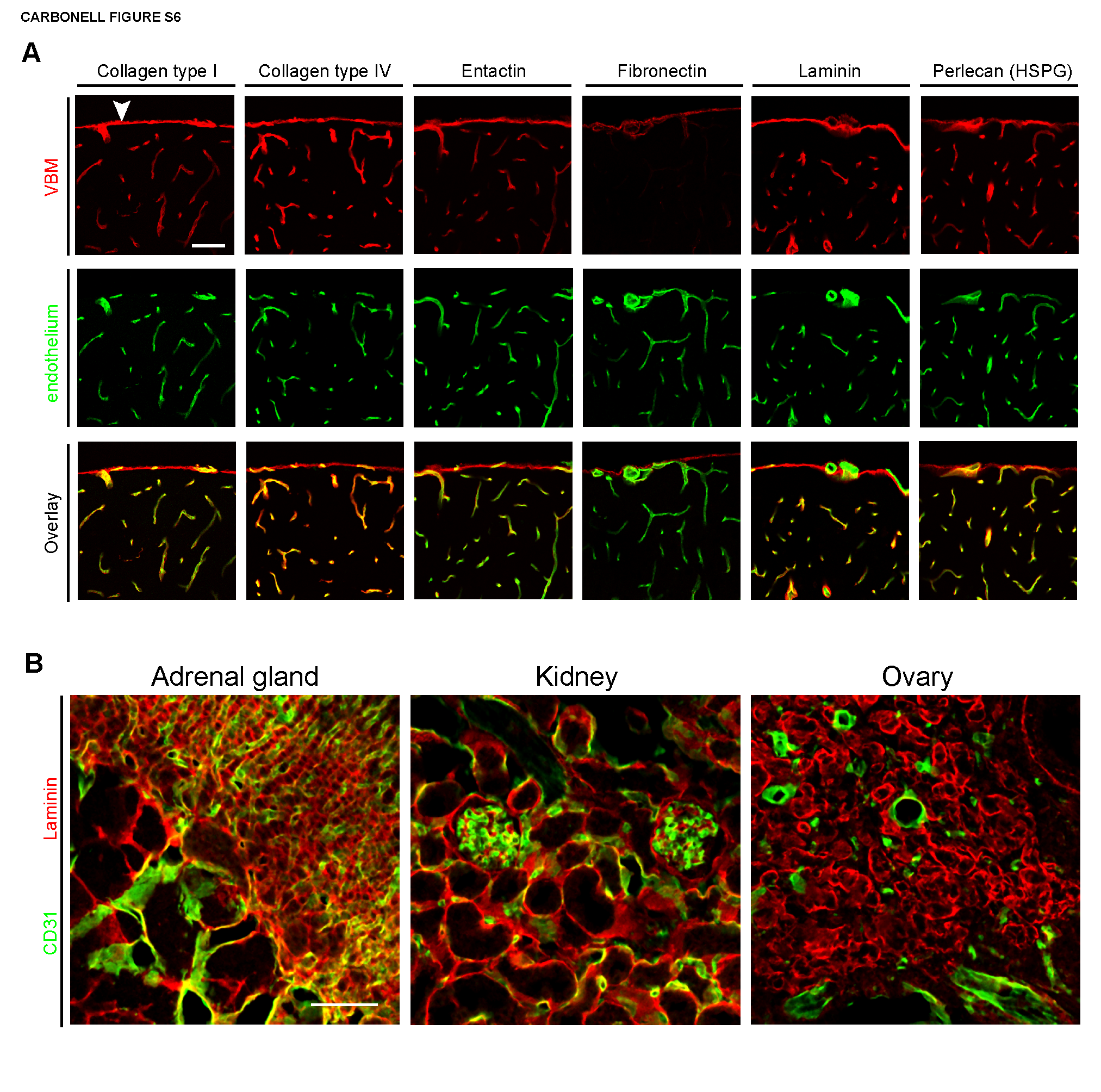

Supplement: Figure S6 — Basement membrane proteins are limited to brain blood vessels (A) Distribution of collagen type I, collagen type IV, entactin, fibronectin, laminin, and perlecan in the normal murine brain was evaluated with immunofluorescence on horizontal sections. Micrographs were acquired in the cortex. The VBM components were largely limited to the vasculature as demonstrated by co-localisation with the endothelial cell markers Glut-1 or CD34 (green). White arrowhead, collagen type I expression in pia mater. Scale bar, 60 µm. (B) In contrast, immunostaining of normal mouse visceral organs (as indicated) produced vascular and extensive extravascular immunoreactivity for laminin. Scale bar, 60 µm. (2.84 MB TIF) [file pone.0005857.s007.tif]

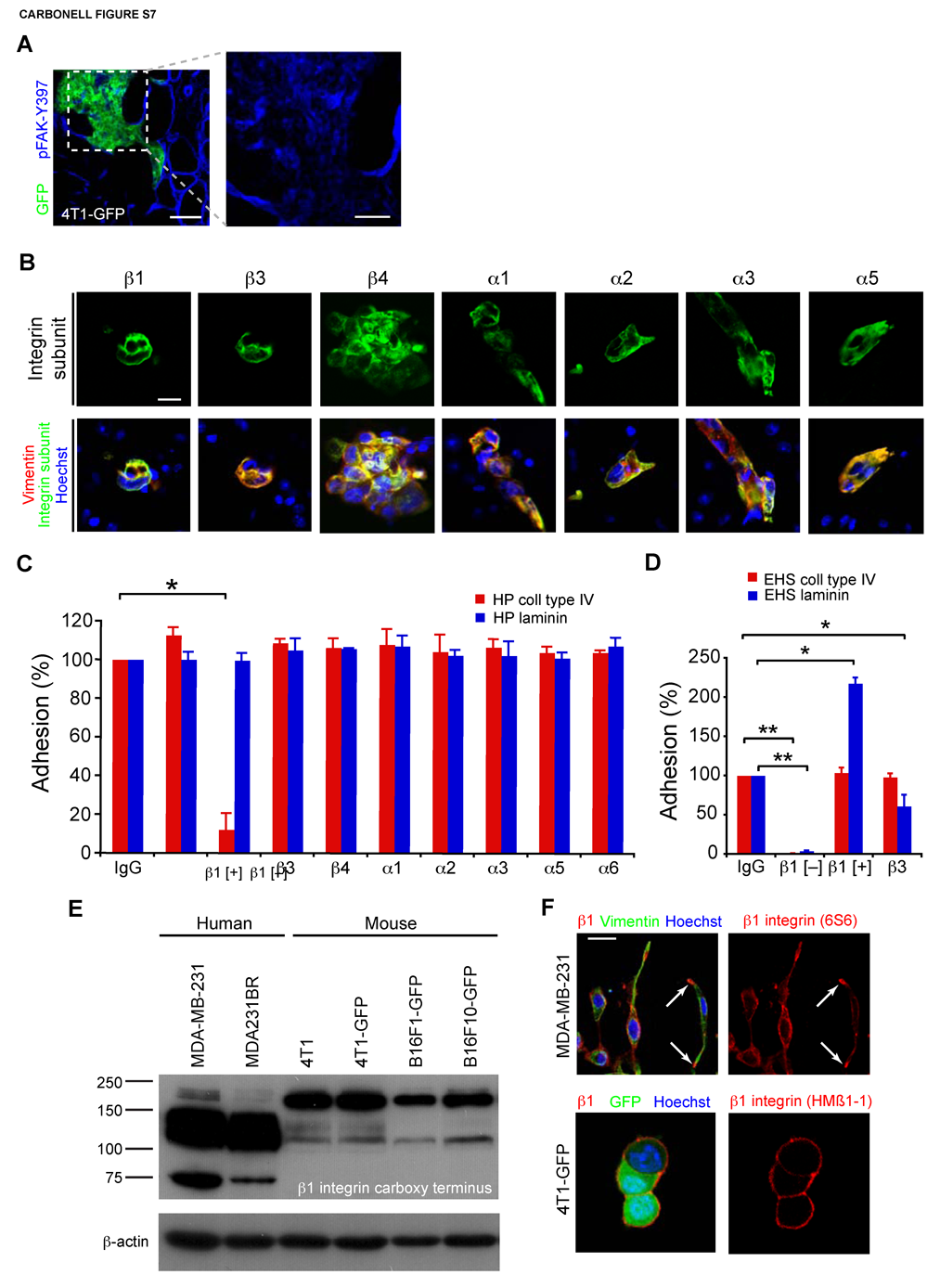

Supplement: Figure S7 — Integrins mediate adhesion to brain vessels. (A) Murine mammary carcinoma 4T1-GFP brain microcolonies are immunoreactive to activated focal adhesion kinase (pFAK-Y397) suggestive of integrin-mediated downstream signaling. Scale bars, 60 µm (left) and 30 µm (right). (B) Expression of integrin subunits previously reported to be expressed in MDA-MB-231 cells in vitro [30] was verified in experimental brain metastases in vivo 7 d after intracardiac injection. All eight subunits (α6, not shown) were readily detectible in microcolonies with indirect immunofluorescence using human-specific monoclonal antibodies. Scale bar, 15 µm. (C) MDA-MB-231 breast carcinoma cells were tested for adhesion to human placental collagen type IV or laminin in the presence of the indicated blocking antibodies (* p>0.01, ANOVA with post-hoc Dunnet multiple comparisons test, performed in quadruplicate and repeated). (D) The effect of blocking antibodies to human β1 or β3 integrin subunits, of control IgG, or of activating β1 integrin subunit antibody was tested on adhesion of MDA-MB-231 human breast carcinoma cells to mouse EHS collagen type IV and laminin (p>*0.01 both comparisons, ANOVA with post-hoc Dunnet multiple comparisons test, performed in triplicate or quadruplicate and repeated). These experiments identified the β1 subunit to be obligatory for MDA-MB-231 breast carcinoma adhesion to murine EHS collagen type IV and laminin and human HP collagen type IV. (E) Western blot for the carboxy terminus of the β1 integrin subunit reveals expression in all tumor cell lines used in this study analyzed (other lines not shown). Numerous glycosylated isoforms [45] as well as species-specific variants can be appreciated. β actin served as loading control. (F) β1 integrin subunit immunofluorescence on the cell membrane in both 4T1-GFP and MDA-MB-231 (as indicated) cells plated in collagen type IV coated wells. Immunoreactivity was also seen particularly distally in cell processes of MDA-MB-231 cells [file pone.0005857.s008.tif]

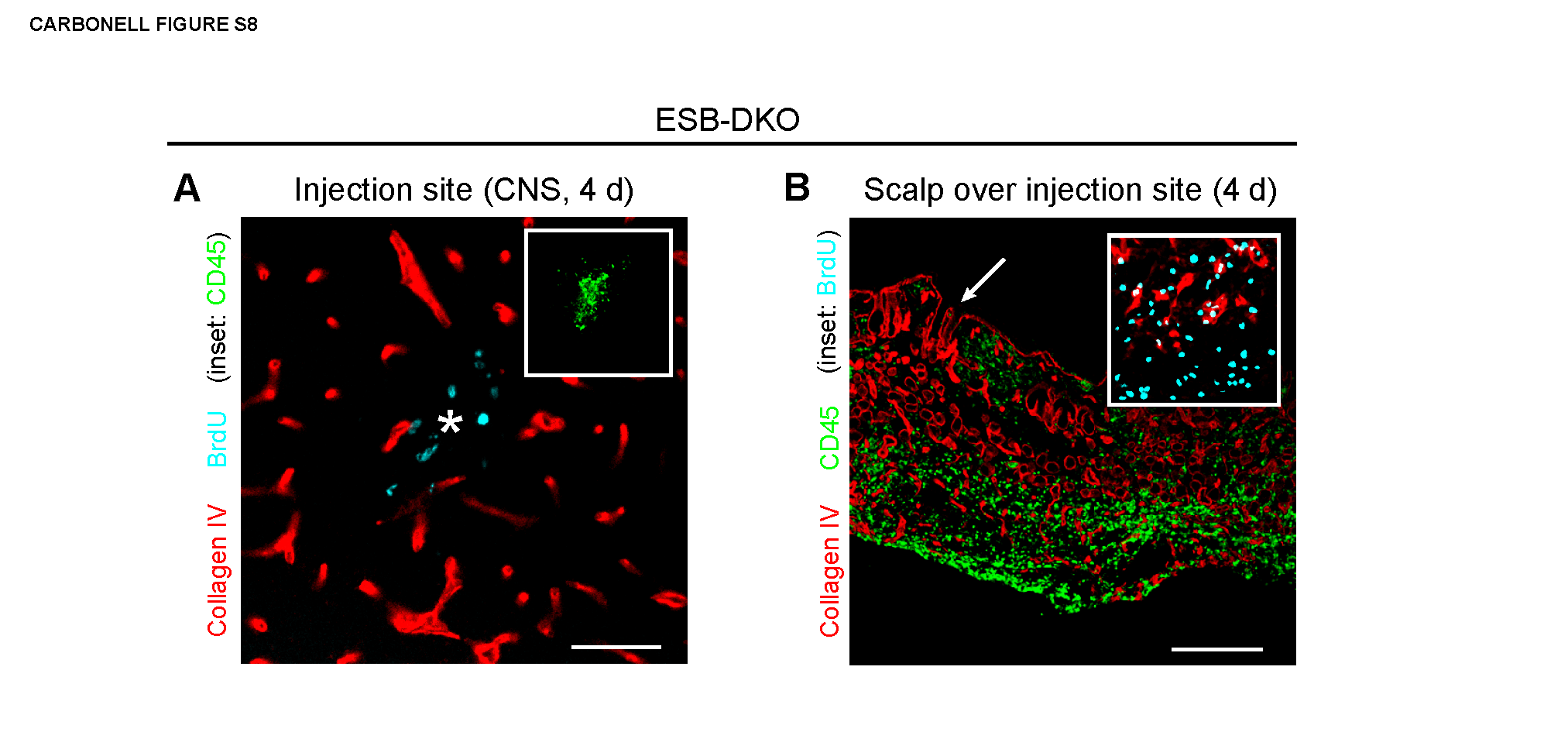

Supplement: Figure S8 — β1 integrin null ESb-DKO cells are not generally growth defective in vivo. (A) BrdU immunohistochemistry (cyan) of tissue sections from DBA/2 mice 4 d after intraparenchymal injection of ESb-DKO cells (green, inset) demonstrates a low baseline of proliferation (horizontal brain section). Scale bar, 30 µm. (B) ESb-DKO cells are able to invade, grow, and proliferate (inset) within several layers of the scalp over the injection site (arrow, hair follicle; coronal brain section). Scale bar, 240 µm. (0.43 MB TIF) [file pone.0005857.s009.tif]
