# Supplementary material for: The Vascular Basement Membrane as “Soil” in Brain Metastasis
Source: PLoS One. 2009 Jun 10;4(6):e5857. doi: 10.1371/journal.pone.0005857 (PMC2689678; doi:10.1371/journal.pone.0005857)
Supplement: Text S1 — Detailed Experimental Procedures (0.14 MB DOC) [file pone.0005857.s010.doc]

**DETAILED EXPERIMENTAL PROCEDURES**

***IN VITRO* EXPERIMENTS**

**Cell lines.** 4T1-GFP, B16F1-GFP, B16F10-GFP, MDA-MB-231, MDA231BR [11], and A7 cells were maintained in DMEM supplemented with 10% FBS and antibiotics. ESb and ESb-DKO cells were maintained as described [32]. K1735M2-GFP cells were maintained in MEM supplemented with 5% FBS, vitamins, non-essential amino acids, sodium pyruvate and antibiotics. GFP-expression was selected with G418 at 250 to 800 µg/ml depending on cell line.

***In vitro* adhesion and proliferation assays.** Adhesion assays were performed in 24- or 96-well pre-coated plates (BD Biocoat) or TC-treated plates coated with varying concentrations of substrate. Media was changed to serum free for 30-60 mins. Plates were blocked with 0.1% BSA (Sigma) for 1 hour and cells were resuspended and plated. For adhesion, non-adherent cells were aspirated after 1 or 2 h and wells gently rinsed and then fixed in 4% paraformaldehyde. Cells were stained with 0.1% crystal violet, dye extracted in 0.5% Triton X-100 and read on a plate reader (Tecan) at 595nm. For proliferation, cells were continuously incubated for 24 to 72 hours and then submitted for either 1) standard MTT assay (Sigma) or 2) colorimetric BrdU ELISA as per manufacturer instructions (Roche). Precipitate was extracted and absorbance read at 570 nm and 370 nm, respectively.

***Ex vivo* brain slice co-culture assay.** Brain slices and *in situ* medium were prepared as described [28,48]. Non-GFP lines were loaded with the vital dye CMRA (Invitrogen) at 10 µM according to manufacturer’s protocol. 5 × 10^3^ tumor cells were plated upon each brain slice and cocultured at 37°C for 2 hours before fixation in 4% paraformaldehyde. Vessels were visualized by vital labeling with 10 µg/ml GS-IB4 isolectin conjugated to either Alexa 488 or 568 (Invitrogen) for 1 hour prior to coculture [28]. Confocal z-stacks were acquired at 20x from the surface of the cultures up to 150 µm into the slice in both the thalamus and cortex. There was no difference in cell behavior observed between these two regions nor was there appreciable invasion into the slices at this timepoint (data not shown). Cells were scored according to morphology. “Mixed” morphology pertains to cells that were neither round nor elongated, but displayed for example filopodia or multipolarity. Elongated cells were defined as bipolar cells with a length at least 2x width or unipolar cells with a dominant protrusion at least 2x the diameter of the nucleus.

***Ex vivo* brain slice invasion assay.** 4T1-GFP spheroids were co-cultured with postnatal mouse brain slices [48-50] for 3 to 7 days before light fixation in 4% paraformaldehyde. Vessels were visualized with immunofluorescence for collagen type IV. Due to high vascular density of brain sections, only spheroids at the edges of each slice were quantitatively analyzed.

**Brain section adhesion assay.** Freshly isolated mouse or human brain tissue (from neurosurgical resection specimens) were snap frozen in liquid nitrogen, cut by cryostat at 10-20 µm, and mounted on glass slides. After rehydration, sections were blocked in 0.1% BSA (Sigma) for 1 hour. 5 × 10^4^ tumor cells were plated and co-cultured for 2 hours followed by 3 rinses in PBS on an orbital shaker. Sections were fixed in 4% paraformaldehyde and processed for fluorescent immunohistochemistry prior to analysis.

***IN VIVO* EXPERIMENTS**

**Experimental brain metastasis models.** All animal procedures were approved by the UK Home Office. Experimental brain metastases were established by intracardiac injection of 10^5^ tumor cells [11]. Alternatively, direct intraparenchymal injection of 5 × 10^3^ to 10^4^ cells was performed into the striatum or hippocampus with a stereotaxic apparatus (Benchmark, MyNeurolab.com) as described [51].

**Spontaneous brain metastasis model.** 10^4^ to 10^5^ 4T1-GFP cells in 10-20 µl PBS were injected into the left 4^th^ mammary fat pad [27]. Animals were sacrificed at 35 to 42 d. Due to frequent loss of GFP expression by tumor cells in late metastases, spontaneous colonies in the CNS were identified by immunohistochemistry for EpCAM (CD236) or cytokeratins which are enriched in carcinomas and largely absent from neural tissues.

***In vivo MR.*** The inability to detect brain metastases which grow by vascular cooption with contrast-enhanced MRI has been demonstrated [52]. MRI imaging was performed with a 7-Tesla horizontal bore magnet with a Varian Inova spectrometer (Varian) between 2 and 14 d post tumor inoculation as described. A series of seven coronal 1mm thick T_2_-weighted images spanning the entire brain, from the olfactory bulbs through to the cerebellum, were acquired using a fast spin-echo sequence (TR=3sec, TE=38ms, field of view = 2.5cm x 2.5cm, matrix = 128 x 125, averages = 2). Subsequently, a series of seven coronal 1mm thick T_1_-weighted images were acquired using a spin-echo sequence (TR=500ms, TE=20ms, field of view = 2.5cm x 2.5cm, matrix = 128 x 128, averages = 4) both prior to and 10 minutes after injection of 40μl of the intravascular contrast agent gadolinium-DTPA-BMA (Gd) to investigate BBB integrity. Following MRI the animals were either recovered for subsequent imaging sessions, perfusion-fixed, or humanely euthanized and organs collected and snap frozen for histopathological assessment. T_2_- and post-Gd T_1_-weighted images were assessed qualitatively for evidence of vasogenic edema and BBB breakdown (contrast enhancement), respectively. For presentation all brains were masked to remove extra-cranial signals.

**Transcranial imaging *in vivo*.** Transcranial imaging was performed up to 3 times via implanted cranial window as described with a Leica TCS-SP2 AOBS confocal microscope equipped with 488, 543, and 633 nm laser lines and a Spectra-Physics MaiTai Ti-Sapphire pulsed laser. Craniectomy was performed over the left hemisphere and dura carefully removed with a finely-hooked 30g needle and microforceps. Care was taken to keep exposed cortical surface moist with saline. Tumor cells were injected stereotaxically as described (coordinates for hippocampus: A/P, centered between lambda and bregma; L, 1 mm; V, 2.5 mm). A semi-round coverglass (6 mm round coverglass cut in half, VWR) was placed over the craniectomy site and secured to the skull with cyanoacrylate gel to produce a water-tight seal. For *in vivo* vascular niche experiments, non-perfused vessels were obtained in two ways: 1) superficial veins directly in the path of the injection needle were severed by the procedure and left *in situ* and 2) superficial veins severed as a direct consequence of dural manipulation and removal were identified and placed directly upon the injection site. Non-perfused vessels were easily identified by the presence of thrombus within the lumens, confocal laser attenuation demarcating the contours of the vessel walls, and only partial or complete lack of dextran filling. Hemostasis was achieved prior to closure with the coverslip and free blood cells gently rinsed away with saline. Prior to imaging, 50-100 µl of 5-10% Rhodamine B- or TRITC-dextran 77 to 150 kD (Sigma) in saline was administered to some animals via tail vein to highlight vessels. Animals were secured in a custom-built low profile mouse head holder and situated on the microscope stage. Temperature was maintained at ~37°C with a ventral heat pad (Harvard Apparatus). Images were acquired with a 10 or 40x aqueous immersion lens (Leica) to a depth of up to ~150 µm in the cortex. An aqueous imaging chamber was created prior to each session by placing a ring of cyanoacrylate gel around the cranial window and filling it with dH_2_O before the gel had a chance to set. This created a temporary but stable aqueous “bubble” necessary for immersion of the microscope objective and obviated the need for cumbersome metal imaging chambers to be permanently affixed to the animal’s skull. Identical recording fields between sessions were identified by vascular anatomy and co-registry of the head holding device with the microscope stage.

**Human tissue.** Surgical tissue surplus to diagnostic requirements and post-mortem tissue was retrieved from the Thomas Willis Oxford Brain Collection with approval from the local research ethics committee (reference 06/Q1604/141). Metastatic tumors were classified according to known primary site or immunohistochemical profile during routine diagnostic work-up. Well-established breast carcinoma metastases were defined as tumors that were surgically resected after a macroscopic mass lesion was identified on neuroimaging. A metastasis was included in the study if the specimen contained at least focally a well-preserved tumor-brain interface. Micrometastases were defined as tumors in the brain parenchyma that were less than 5 mm in diameter and did not contain a central solid sheet of cells. Carcinomatous metastases were those associated with carcinomatous meningitis. Micrometastases and the parenchymal component of carcinomatous growth were considered to reflect early stages of invasion. Tumor cells were immunostained with Cam5.2 antibody (monoclonal, 1:10, BD) and endothelial cells with QBend 10 (CD34) (monoclonal, 1:200, Dako Cytomation). All immunohistochemical assays were carried out using the Dako Envision kit on routine paraffin-processed tissue. Sections were reviewed and digitally photographed with an Olympus BX51 microscope. Angiotropic invasion was defined as perivascular tumor cell growth at the tumor-brain interface. This was divided into single-cell invasion or collective (bulk) invasion (two or more cell layers thick). Angiotropic growth in cases with carcinomatous growth or micrometastases was further quantified in Cam5.2-stained sections by counting in consecutive microscopic fields the tumor cell profiles that were in a perivascular location compared to those that were not clearly vessel-associated. A maximum of 1000 cells were counted per lesion. Vascular changes at the tumor-brain interface were also recorded. Endothelial hyperplasia (more than a single layer of lining endothelium) with or without vascular splitting (an endothelial bridge within a vessel) and microvascular proliferation (formation of atypical vascular channels, e.g. glomeruloid structures) were recorded as definitive morphological evidence of angiogenesis. Cryosections of snap-frozen, mildly gliotic but otherwise normal temporal neocortex from partial lobectomy specimens (*n* = 3) were used for *in vitro* adhesion co-culture assays.

**Integrin inhibition studies.** Cells were placed in serum free media for 30-60 min. After trypsinization and resuspension, 5 to 20 µg/ml of function modulating monoclonal antibodies against integrin subunits or isotype matched control antibodies were added to the cells, the mixture vortexed, and then was placed on ice for 30-60 min prior to use. The following functional anti-human integrin subunit antibodies (Millipore) were used in this study (as verified in [53]): anti-β1, clones 6S6 and 21C8 (blocking and non-blocking, respectively); anti-β3, clone B3A; anti-β4, clone ASC-3; anti-α1, clone FB12; anti-α2, clone P1E6; anti-α3, clone ASC-1; anti-α5, clone P1D6; and anti-α6, clone NK1-GoH3. For the *in vivo* assays, MDA-MB-231 breast carcinoma cells (10^4^) were pre-incubated in 20 µg/ml of the indicated antibody on ice for 30m as described above. Cells and antibody were then injected into the striatum in a volume of 2 µl. No further antibody was administered to mice for the duration of the experiment.

**Histology.** Tissue was collected under terminal anesthesia after transcardiac perfusion with saline and 4% paraformaldehyde or organs were freshly isolated and snap frozen or immersion fixed. Immunohistochemistry was performed as described in 15-30 µm cryostat sections as described. Proliferation was assessed in some animals with intraperitoneal injection of 1 mg BrdU (Sigma) at least 2 hours prior to sacrifice.

**Vascular Association.** Vascular association of intraparenchymal tumor microcolonies was determined between 3 and 14 d after intracardiac inoculation of tumor cells (see above). After sacrifice and brain isolation, the cerebellum was blocked and the entire forebrain (tips of olfactory bulbs to the tectal plate; measuring approximately 10 mm anterior/posterior) was submitted to continuous serial cryostat sectioning at 15 µm. Sections were mounted on Fisher Superfrost Plus slides (Fisher Scientific) and processed with immunohistochemistry for vessels and tumor cells (see below). Quantitation was performed on a uniform sampling of 12 sections from each brain approximately 500 µm apart and roughly corresponding to the following plates from the C57BL/6J coronal atlas from the Mouse Brain Library [http://www.mbl.org]: 3, 5, 7, 9, 11, 13, 15, 17, 19, 21, 23, and 25. The entire sections were scanned at 10 and 40x for tumor profiles, the relationship to vessels noted, and images captured with confocal microscopy.

**Histological quantitation.** (1) Tumor area was calculated from confocal images taken at 20 to 40x. The tumor was outlined with the polygonal draw function in ImageJ and area in pixels was measured and converted into microns in MS Excel. (2) Vascular density was calculated from confocal images taken at 40x. The number of vascular profiles based on Glut-1 IHC per field was counted. (3) The distance of BrdU positive profiles to nearest vessels were measured in 40x fields. For all analyses, between 3 to 6 brain sections separated by at least 100 µm were measured for each animal and tumor area averaged for each mouse.

**Immunohistochemistry.** Fluorescence immunohistochemistry was performed with either standard indirect technique (fluorophore-conjugated secondary antibodies; Invitrogen) or with tyramide signal amplification (TSA). TSA kits from Perkin Elmer were used as recommended by the manufacturer, varying the tyramide reagent from 1:50 to 1:200. M.O.M. blocking reagent (Vector Labs) was used for murine monoclonal antibodies used on mouse tissue or alternatively directly conjugated antibodies were made with the Zenon mouse IgG labeling kits (Invitrogen). The following primary antibodies were used in this study for fluorescence immunohistochemistry/biochemistry in experimental tissues:

| **Target** | **Host** | **Vendor** | **Item #** | **Use/**  **Conc** | **Staining technique** | **Notes** |
| --- | --- | --- | --- | --- | --- | --- |
| BrdU | sheep | Abcam | ab2284 | FIHC 1:100 | Abcam protocol, TSA | biotin conjugated |
| CD31 | rat | Abcam | ab22529 | FIHC 1:50 | TSA |  |
| CD31 | rat | Biolegend | 102508 | FIHC | TSA | Clone MEC13.3 |
| CD34 | rat | Abcam | ab8158 | FIHC 1:50-100 | TSA |  |
| CD45 | rat | BD Pharmingen | 550539 | FIHC 1:100 | TSA |  |
| CD326/EpCAM | rat | Biolegend | 118202 | FIHC 1:100 | TSA |  |
| collagen type I | rabbit | Abcam | ab34710 | FIHC 1:250 | indirect |  |
| collagen type IV | rabbit | Abcam | ab19808 | FIHC 1:250-1000 | indirect, TSA | +/- pepsin |
| collagen type IV | goat | Southern Biotech | 1340-01 | FIHC 1:250-1000 | indirect, TSA | +/- pepsin |
| collagen type IV | mouse | Sigma | C1926 | FIHC 1:100-250 | indirect | +/- pepsin |
| cytokeratins (pan) | mouse | Sigma | C2562 | FIHC 1:100 | indirect |  |
| entactin | rat | Abcam | ab44944 | FIHC 1:250 | TSA | clone ELM1 |
| fibronectin | rabbit | Abcam | ab23750 | FIHC 1:100 | indirect |  |
| GFP | goat | Abcam | ab6662 | FIHC 1:250 | direct | FITC conjugated |
| GFP | chicken | Abcam | ab13970 | FIHC 1:500-1000 | indirect |  |
| glut-1 | rabbit | Millipore | AB1340 | FIHC 1:500 | indirect |  |
| HSPG2/perlecan | rat | Abcam | Ab17848 | FIHC 1:100 | TSA | clone SPM225, prediluted |
| integrin alpha1 | mouse | Millipore | MAB1973Z | FIHC 1:100 | indirect | clone FB12 |
| integrin alpha2 | mouse | Millipore | MAB1950Z | FIHC 1:100 | indirect | clone P1E6 |
| integrin alpha3 | mouse | Millipore | MAB2058Z | FIHC 1:100 | indirect | clone ASC-1 |
| integrin alpha5 | mouse | Millipore | MAB1956Z | FIHC 1:100 | indirect | clone P1D6 |
| integrin alpha6 | rat | Millipore | MAB1378 | FIHC 1:100 | TSA | clone NKI-GoH3 |
| integrin beta1 | rabbit | Abcam | ab52971 | FIHC 1:100  WB 1:1000 | TSA |  |
| integrin beta1 | mouse | Millipore | MAB2253Z | FIHC 1:100 | indirect, TSA | clone 6S6 |
| integrin beta1 | mouse | Millipore | MAB2250Z | FIHC 1:100 | indirect | clone 21C8 |
| integrin beta1 | hamster | Biolegend | 102201 | FIHC 1:100 | indirect, TSA | clone HMβ1-1 |
| integrin beta3 | mouse | Millipore | MAB2023Z | FIHC 1:100 | indirect | clone B3A |
| integrin beta3 | hamster | Biolegend | 104310 | FIHC 1:100 | TSA |  |
| integrin beta4 | mouse | Millipore | MAB2058Z | FIHC 1:100 | indirect | clone ASC-3 |
| laminin | chicken | Abcam | ab14055 | FIHC 1:250-1000 | indirect | +/- pepsin |
| laminin1+2 | rabbit | Abcam | ab7463 | FIHC 1:500 | indirect, TSA | +/- pepsin |
| laminin | rabbit | Sigma | L9393 | FIHC 1:250 | Indirect | +/- pepsin |
| vimentin | rabbit | Vector Labs | VP-RM17 | FIHC 1:100 | indirect, TSA |  |
| ZO-1 | rabbit | Invitrogen | 40-2200 | FIHC 1:250 | Indirect |  |

FIHC, fluorescent immunohistochemistry; TSA, tyramide signal amplification; WB, western blot
